# Public mass shootings cause large surges in Americans’ engagement with gun policy

**DOI:** 10.1093/pnasnexus/pgad407

**Published:** 2023-11-29

**Authors:** Tyler T Reny, Benjamin J Newman, John B Holbein, Hans J G Hassell

**Affiliations:** Department of Politics, Claremont Graduate University, Claremont, CA 91711, USA; School of Public Policy and Department of Political Science, University of California, Riverside, CA 92521, USA; Frank Batten School of Leadership and Public Policy, University of Virginia, Charlottesville, VA 22903, USA; Department of Political Science, Florida State University, Tallahassee, FL 32306, USA

**Keywords:** mass shootings, gun violence, political behavior, democracy, regression discontinuity

## Abstract

As public mass shootings continue to plague the United States, a growing scholarly literature seeks to understand the political effects of these tragic events. This literature, however, focuses on public opinion or turnout and vote choice, leaving open to question whether or not public mass shootings affect a range of other important actions citizens may take to engage with gun policy. Leveraging the as-good-as random timing of high-publicity public mass shootings over the past decade and an immense array of publicly available and proprietary data, we demonstrate that these events consistently cause surges in public engagement with gun policy—including internet searches, streaming documentaries, discussion on social media, signing petitions, and donating to political action committees. Importantly, we document the behaviors where shootings induce polarizing upswings in engagement and those where upswings skew toward gun control. Finally, we demonstrate that low-publicity shootings largely exert little-to-no effect on our outcomes.

**Significance Statement**

The devastating impact of public mass shootings in America is exacerbated by a lack of policy reform to address gun violence. Why does this inaction occur? One assumption is that, while prompting short-lived emotional reactions, public mass shootings fail to propel Americans to political action. Using an unprecedented amount of proprietary and publicly available data on previously unexplored forms of public engagement with gun policy, we show, contrary to common assumptions, that public mass shootings increase public engagement across a range of political behaviors. Sometimes responses are polarized (increased activity supporting gun control and gun rights), but often shooting-induced responses skew toward gun control. Our results provide causal evidence that Americans respond to acute gun violence with political action.

Every year over the past decade the United States has experienced one or more public mass shooting, where an assailant with firearms entered a public space (e.g. school, religious institution, workplace, shopping venue, or festival) and opened fire on victims in a haphazard manner. These instances of acute gun violence have occurred in every region of the country and have taken the lives of individuals across the spectrum of age, gender, race and ethnicity, religion, and socioeconomic status. Striking, however, has been the lack of sweeping policy change in the United States to curb gun violence given the recurrence of public mass shootings (1, 2). At the federal level, consequential changes to gun law are seldom proposed and rarely make it past the Congressional committee stage (3). What is more, research at the state level finds that, rather than tightening gun restrictions, firearm laws across the 50 states become *less* restrictive following public mass shootings (4).

A common explanation offered by pundits and journalists for the lack of drastic policy change toward greater gun control in response to public mass shootings is that these events, while horrendous, do not propel the American public into action aimed at curbing gun violence (5, 6). For example, a recent editorial in the *Washington Post* asserted, “rarely do Americans who support gun control make it their top priority” (7). Some contend that the persistence of mass shootings has acclimated the American people to gun violence to such an extent that “mass shootings have become white noise” (8), with the result being “Americans get apathetic about gun control” (9). Indeed, journalists have suggested that the recurrence of chaotic events like mass shootings can lead to “crisis fatigue,” which causes society to “collectively throw up our hands and give up on civic engagement” (10). Adding to this, many claim there exists an “enthusiasm gap” about gun policy in America, with opponents of gun control holding gun rights as central to their political identity and highly willing to engage in routine political action in support of their views, while public support for gun control ebbs and flows in a fleeting manner around instances of acute gun violence (5, 11, 12). Topping this off is evidence that many public mass shootings fail to trigger national media attention (9), while the ones that do often succumb to the “issue-attention cycle” (13), whereby spikes in coverage precipitously decline a few weeks after a shooting and the focus of the media—and thus the general public—shifts from gun control to other issues.

Even accounts of gun politics in the United States highlighting the presence of episodes of action toward gun control leave unclear how ordinary citizens engage with gun policy in the wake of gun violence. For example, one long-standing and prominent account depicts gun politics in America as a recurrent cycle of “outrage-action-reaction” resulting in policy gridlock (14). According to this account, instances of horrific gun violence, such as public mass shootings, cause surges in public outrage and political action to achieve gun control which are promptly countered and stymied by powerful gun rights advocates. This account implies that public outrage ebbs and flows around acute incidents of gun violence. Moreover, this account largely documents the “action” stage as comprised of the activities of activist groups and major gun control advocacy organizations (e.g. the Brady Campaign and Everytown for Gun Safety), with little discussion or empirical assessment of mass political behavior and large-scale actions by ordinary citizens. The components of this account best supported by extant empirical research are “outrage” in response to public mass shootings (15) and the power of pro-gun interests groups in shaping legislator behavior and policy outcomes (16–18). In sum, the standing wisdom is that public mass shootings, at best, result in small and ephemeral shifts in *attitudinal* support for gun control that are ultimately unsupported by surges in mass political action aimed at curbing gun violence. And at worst, public mass shootings are part of a constant background noise of ongoing crises for Americans that do not provoke engagement with gun policy.

Is the standing wisdom true? Do Americans, in fact, fail to engage in action to promote gun control following public mass shootings? Moreover, is there a stronger response following public mass shootings among opponents of gun control in defense of gun rights? Surprisingly, existing research does not provide clear answers these questions. On the one hand, in contrast to the claim that mass shootings have become “white noise,” existing research demonstrates that public mass shootings take a significant psychological and emotional toll on the American people (15, 19). On the other hand, looking beyond Americans’ well-being to their political attitudes, past research finds that public mass shootings do not elicit a clear or consistent effect on Americans’ opinions on gun control (20–23). Perhaps more importantly, with respect to electoral behavior, recent research demonstrates that public mass shootings do *not* heighten voter turnout or influence party choice in federal, state, or local elections (24, 25). Judging by these findings alone, one may suspect the standing wisdom is true.

However, conspicuously absent from the literature is research analyzing Americans’ responses to public mass shootings focusing on the myriad ways people may engage with politics and attempt to influence public policy *beyond* reporting opinions to pollsters or casting votes in elections. Following public mass shootings, do Americans do things like seek out political information, engage in political discussion, express their opinions through the display of political banners or flags, sign petitions sent to policymakers, or donate money to policy advocacy organizations? Such behaviors are critically important because public engagement and action around an issue in these ways has a powerful effect on policy above and beyond merely holding an opinion (26–29). Moreover, the opinions individuals profess to hold do not always match the actions that they take, especially when such actions require costs that individuals may not be willing to meet (30, 31). And yet, despite the vital importance of such actions on democratic responsiveness, the scholarly literature renders us without an answer to this question.

Prior research on mass shootings (20, 23, 24) theoretically draws on the literature on “focusing events” (32, 33), which argues that sudden and harmful events forcibly direct societal attention to a problem and mobilize the general public into action to remedy aspects of the status-quo policy environment deemed responsible for the event. Applied to mass shootings, the operative hypothesis is that these appalling events swiftly direct Americans’ attention to gun violence, highlight the problem of inadequate government regulation of deadly firearms, and mobilize the public around policy change toward greater gun control. While the application of this framework to mass shootings has rendered mixed or unsupportive results with respect to Americans’ attitudinal support for gun control or turnout and vote choice in elections, it remains to be seen whether or not this framework finds empirical support when analyzing *nonelectoral* forms of engagement with gun policy. One recent piece of suggestive evidence in support of this framework comes from Goss and Lacombe (34), whose presentation of trends in the volume of letters sent by citizens to the editors of four newspapers illustrate observable spikes in the number of gun-related letters following several high-profile public mass shootings (e.g. Columbine, Sandy Hook, and Parkland). While important in their own right, the authors admit that their findings are descriptive in nature, focus on a single behavior, and do not involve a systematic analysis of the causal effect of a broad set of public mass shootings on gun-related letters to editors. As such, it remains open to question whether public mass shootings cause consistent surges in various forms of behavioral engagement with gun policy.

In this article, we analyze an array of previously unexplored actions Americans may take in response to public mass shootings. We collected a compilation of large-scale publicly available and proprietary data measuring an array of indicators of engagement with gun policy. These indicators include: measures of political information-seeking, such as internet searches for firearm-related policy positions and advocacy organizations (Google Trends data) and streaming prominent documentaries about gun policy (proprietary data from media companies); measures of political discussion and expression, including online political speech (Twitter data) and purchases of pro-gun political flags for display (Amazon.com sales data); and finally, measures of efforts to directly influence politicians and public policy, such as signing petitions sent to lawmakers (Change.org and Patriot Voices data) and donating money to the political action committees (PACs) of gun policy advocacy organizations (Federal Election Commission contributions data). These measures capture forms of engagement that vary in their costs in time, effort, and money to ordinary citizens, as well as in their potential visibility and significance to key policy actors. Importantly, for most of these indicators of engagement, we measure activity on both sides of the gun policy debate—that is, activity oriented toward gun control and gun rights. This enables our analyses to speak to the issue of *countervailing* political engagement, with the overarching goal being the detection of a potential tilt in activity toward one side of the gun policy debate vs. the other.

With these data in hand, we leverage the as-good-as random timing of public mass shootings occurring over the past decade to estimate the causal effect of these events on Americans’ engagement with gun policy. Given known variation in media coverage of public mass shootings (35), the importance of mass media in shaping the political effects of local events by elevating their salience (36, 37), and the considerable power of the media in general to shape political priorities and discourse (38, 39), we collected information about the level of national news media attention given to each public mass shooting. The principal expectation is that receiving extensive publicity elevates a shooting to the status of “focusing event” capable of mobilizing public engagement with gun policy, whereas shootings attracting less national media attention will remain localized events with limited impact on mass political behavior.

In contrast to the standing wisdom that public mass shootings do not propel Americans into action, our findings demonstrate that *high-publicity* public mass shootings cause drastic increases in internet searches for gun control and gun control advocacy organizations, online political speech mentioning gun control and gun control advocacy organizations, signing petitions demanding gun control, and donations to the PACs of gun control organizations. These spikes in political activity are typically quite large, constituting a multiple SD shift from preshooting patterns for many of these outcomes. Interestingly, high-publicity public mass shootings typically prompt countervailing spikes in information-seeking about and online discussion of gun rights and pro-gun political organizations. That said, when analyzing sales on Amazon.com of popular political flags with gun rights slogans, we fail to observe any effect of public mass shootings on purchases. Adding to this, when turning our focus to efforts by Americans to directly influence lawmakers and policy advocacy organizations—namely, petition signing and PAC donations—we find that high-publicity public mass shootings only trigger activity oriented toward gun control. Finally, we find that *low-publicity* public mass shootings typically fail to instigate significant changes in Americans’ level of engagement with gun policy.

## Beyond opinions and voting: measuring Americans’ engagement with gun policy

Our analysis relies on a unique and immense compilation of publicly available and proprietary data on *daily* indicators of Americans’ engagement with gun policy. The various datasets used in our analysis are summarized in Table 1. The data we compiled far surpass previously published work in terms of breadth and granularity.

**Table 1.** Data sources.

Political act	Data sources	Date range	Examples	Shooting events
*Information-seeking*				
Internet Searches	Google Trends	2011 Jul. 6–2022 Feb. 26	“Gun Control,” “Gun Rights,” “Brady Campaign,” “NRA,” “Gun Owners of America”	40

Documentary Streams	PBS Frontline, The Guardian, Real Stories	2015 Jan. 6–2021 Sept. 8	*Gunned Down: Power of the NRA*, *NRA Under Fire*, *Gun Nation*, *The Gun Store*	28

*Discussion & Expression*				
Social Media Posts	Twitter	2011 Apr. 29–2021 Jun. 30	“#guncontrol,” “#gunrights,” “#everytown,” “#NRA”	37

Flag Purchases	ANLEY INC.	2021 Jan. 1–2022 Jan. 31	“Second Amendment,” “Liberty or Death,” “Come and Take It”	3

*Influencing decision-making*				
Petition Signing	Change.org, Patriot Voices	2013 Mar. 6–2023 May 12	“Pass Common Sense Gun Control,” “Stop the sale of guns at Walmart Stores,” “Physicians Demand Stricter Gun Control,” “Maryland Carry Laws Prevent Citizens From Legally Protecting Themselves,” “Defend Second Amendment”	32

Donating to PACs	FEC	2013 Jan. 7–2020 Dec. 31	Giffords PAC, NRA Victory Fund	27

We begin with daily activities in pursuit of information about gun policy—namely, internet search behavior and viewership of documentaries. For internet search behavior, we retrieved publicly available data on search histories from Google Trends between 2011 July 6 and 2022 February 26. Because of the private nature of internet searches, such information represents true interests (40), can be used effectively to identify information acquisition among individuals in an area (40–43), and has been shown to match actual behaviors and outcomes (44–47). We collected Google Trends data on Americans’ internet searches about basic gun policy positions (“Gun Control” and “Gun Rights”) and political organizations working to achieve gun control (“Brady Campaign” and “Everytown for Gun Safety”) and to preserve gun rights (“the NRA” and “Gun Owners of America”). We combine these with data on daily searches for terms unrelated to gun violence and gun policy (e.g. “Recycling”), which we use to perform placebo tests with the expectation of null effects of public mass shootings on these presumed treatment-irrelevant outcomes.

For additional self-educating behavior, we queried Google’s videos tab for “gun control documentary” and “gun rights documentary.” We contacted the media companies who produced the films in the search returns with requests for daily streaming data on the company’s proprietary web platform and/or their YouTube videos page. Most staff within media companies are guarded from the public—making it difficult to contact relevant personnel with data requests—and the majority of our requests did not receive a response or were denied. In total, we received proprietary daily streaming data for four popular documentary films about gun politics in America. First, we received data for one of the most popular American gun politics documentaries released within the past decade: *Gun Nation*, produced by the British newspaper *The Guardian*.^a^ This documentary was released in 2016 September 16 and had been streamed over 490,000 times at the time of our data collection. We received streaming data for this documentary from *The Guardian’s* YouTube channel starting in 2016 September 16. Accompanying this, the American Public Broadcasting Service (PBS) provided us with data for two publicly available documentaries produced by their investigative journalism program *Frontline*. The first documentary, *Gunned Down: The Power of the NRA*, was released in 2015 January 6 (streamed over 461,000 times at the time of data collection), and the second, *NRA Under Fire*, was released in 2020 March 24 (and had been streamed over 45,000 times). Finally, we received YouTube streaming data for the *Real Stories* documentary, *The Gun Store*, a 2019 gun violence documentary that had been streamed over 13,000 times at the time of data collection. For each documentary, we received daily streaming data from their release date up through between June and September 2021. We make no claim that these four documentaries are representative of the universe of extant documentary films about gun policy in America. Rather, we began with a purposive sample of popular documentary films based on Google search returns and the resulting four documentaries we analyze represent a convenience sample of retrieved data. Despite this, our sample includes popular documentaries released by prominent media outlets and provides us with a meaningful first step in understanding the impact of public mass shootings on efforts by Americans to educate themselves about gun policy via streaming documentaries.

We complement these indicators of daily information-seeking with data on the daily volume of online discussion of gun policy on the social media platform Twitter. Discussion of political issues on platforms like Twitter is a common form of contemporary political activity (48). Twitter reported having over 69 million monthly active users in 2021,^b^ and roughly 23% of American adults in 2021 reported using Twitter.^c^ Beyond providing a space where citizens discuss political issues, Twitter also serves as an arena where the public interacts with government officials, resulting in the demonstrated capacity of discourse on Twitter to direct the attention of legislators and shape their political agendas (26). Twitter data have been used to understand a variety of social and political phenomena (26, 49–51), including specifically measuring public discussion of political issues (38). We used Brandwatch’s Crimson Hexagon to collect the daily count of tweets from April 2011 to June 2021 that included gun policy position hashtags (“#guncontrol” and “#gunrights”), mentioned gun policy advocacy organizations (“#everytown” and “#NRA”), or an unrelated hashtag used as a placebo (“#recycling”). All in all, our dataset totals nearly 20 million tweets (*n*=19,877,924).

In addition to posting comments on social media, a common way Americans express their views is displaying political signs or flags (52). Prior research finds that displaying political signs can exert modest yet reliable persuasion effects on nearby residents (53) and that an estimated 12 to 19% of Americans have displayed a political sign at their home.^d^ Applied to gun policy, people may display signs or flags that convey their support or opposition to gun control. A search of the Amazon.com marketplace led us to identify three popular flags endorsing gun rights sold by one of the largest online vendors of political flags, ANLEY INC. These three flags are: (i) a “Second Amendment” American flag displaying text of the second amendment, (ii) a “Liberty or Death—2nd Amendment” flag displaying a skull and crossbones with rifles, and (iii) a “Come and Take It” flag exhibiting a rifle. We contacted ANLEY INC. and the CEO of the company provided us with daily sales data for each of these flags from the company’s store on Amazon.com between 2021 January 1 and 2022 January 1. Using these data, we constructed a series of the daily count of sales for each flag and for all three flags combined (*n*=46,276 total sales during this time period). While we were able to locate flags endorsing gun rights, there is a notable absence of flags endorsing gun control. ANLEY does not carry a single flag endorsing gun safety or control, and a search on Amazon.com for “gun control flag” did not render a single result. As such, our analysis of flag sales offers a window into the effect of public mass shootings on a behavior seemingly unique to the gun rights side of the policy debate.

To extend our analysis to act directly intended to influence lawmakers and public policy, we obtained data on daily petition signing and political contributions to gun policy advocacy organizations. For petition signing, we submitted requests to the creators of popular gun policy petitions on *Change.org*, one of the largest and most popular websites for creating and circulating political petitions in the world. There are over 200 petitions at Change.org categorized under the topic heading “gun control” and the petition bearing the most signatures, titled “Pass Common Sense Gun Control,” was created by one of the students surviving the 2018 Stoneman Douglas High School shooting. We submitted a request for data to the creator of this petition, who subsequently shared anonymized data on all signatures along with the date and country of residence of the signer. The data included n=375,032 signatures from the United States collected between February 15, 2018 and July 20, 2021. We accompany this with similarly anonymized signature data from the second most popular gun control petition on Change.org, which was created by a physician in San Francisco, CA, on October 2, 2017, following the mass shooting in Las Vegas. This petition, entitled “Physicians Demand Stricter Gun Control,” included n=220,003 signatures collected in the United States between October 3, 2017 and May 12, 2023. The third most popular petition on Change.org was created by a Walmart employee following the mass shooting at a Walmart store in El Paso, TX, on August 3, 2019. This petition, entitled “Stop the sale of guns at Walmart stores,” included n=163,457 signatures collected in the United States between August 6, 2019 and August 16, 2021. In total, we have data on three of the most popular gun-related petitions on Change.org, which combined include over three quarters of a million signatures.

We contrast data on these two prominent gun control petitions with data on the most popular gun rights petition we were able to locate on Change.org. This gun rights petition, entitled “Maryland Carry Laws Prevent Citizens From Legally Protecting Themselves,” was directed at the Governor of Maryland and demanded more permissive gun laws in the state. The reach of this petition is far more modest than the two gun control petitions we analyze, with n=37,765 signatures in the United States collected between November 16, 2015 and December 19, 2017, when the petition was closed. While the petition is specific to a single state, it drew nationwide attention and was signed by residents of all 50 states. In addition to this gun rights petition on Change.org, we scraped the daily signatures to a Patriotvoices.com petition on defending the 2nd Amendment. *Patriot Voices* is a petition-based organization founded by Rick Santorum to fight for conservative issues in Washington D.C. The petition has gathered n=4,344 signatures since 2013, when it was established. We transformed the raw signature data for these four petitions into daily counts of signatures. We should note that while these petitions do not represent a random sample of the universe of extant gun policy petitions, or even those housed on a platform like Change.org, they are a purposive sample in that they are among the most popular online petitions on each side of the gun policy debate and three of these petitions are listed on one of the most popular online petition platforms. As such, analysis of these petitions provide important initial insight as to whether public mass shootings propel Americans to engage with gun policy by signing prominent online petitions.^e^

Finally, we accompany the petition data with information retrieved from the United States Federal Election Commission (FEC) on publicly reorted political contributions made between January 7, 2013 and December 31, 2020 to the two largest gun control and gun rights PACs operating in the United States that were actively raising money during this time period—the Giffords PAC (gun control) and the National Rifle Association (NRA) Victory Fund (gun rights).^f^ Relying on donations to PACs likely yields a substantial under-count of donation activities to these organizations but itemized donations data to 501(c)3 and 501(c)4 gun policy nonprofits, which likely raise the vast majority of money following mass shootings, is not publicly available—a long acknowledged fact in previous research that works with donations data (54).

## Analytic strategy

To identify the effect of public mass shootings on our outcomes, we use a regression discontinuity in time (RDiT) approach. Regression discontinuity designs (RDDs) leverage as-good-as-random variation and continuity in potential outcomes around an arbitrary cutoff to estimate a causal treatment effect (55–59). RDDs have been shown to benchmark well to randomized control trials, e.g. (60). The RDD that we use rests on the reasonable assumption that the precise timing of mass shootings are unanticipated by the public and are exogenous to the mass behaviors that we consider as outcome variables.^g^

**Fig. 1.**
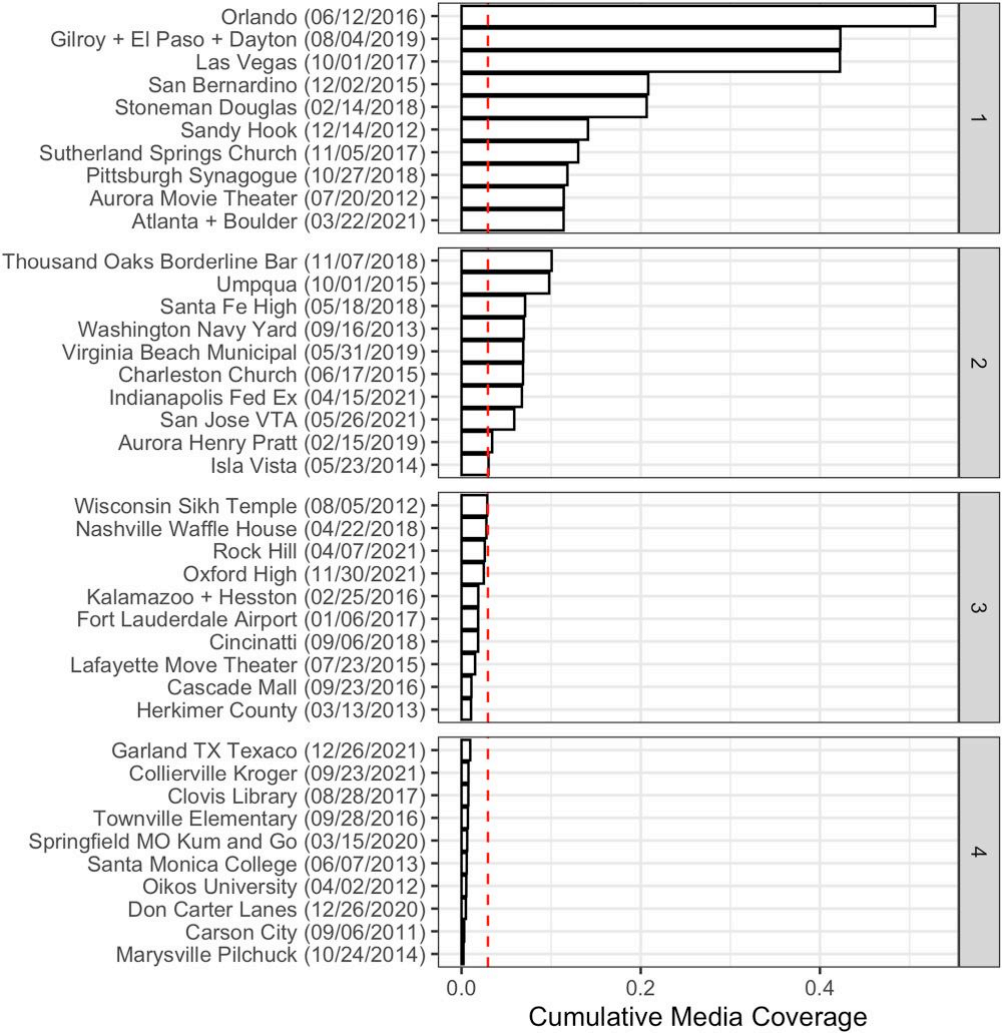
National media attention to public mass shootings, 2011 to 2021. Bars indicate cumulative proportion of media coverage collected by Media Cloud (top-50 news sources in the United States) that mentioned the terms “mass shooting” for the week following each shooting. Vertical dashed line indicates median cumulative coverage. Source: Media Cloud.

Our analysis focuses on 44 public mass shootings occurred between 2011 and 2021. These 44 shootings are listed in Fig. 1 and Appendix Table A1. The term “mass shooting” entails a broad umbrella of events typically involving three or more fatalities (not including the shooter) in a single shooting event.^h^ Public mass shootings are a subset of mass shootings involving an assailant with firearms entering a public space and opening fire on victims in a haphazard manner. Excluded from this subset are mass shootings occurring in: private homes and other residential settings targeting family members, spouses and romantic partners; public spaces resulting from spontaneous altercations between belligerents carrying firearms (e.g. bar fights); and public spaces resulting from criminal or gang activities (e.g. drive-by shootings or police shootouts). Much of the social science research analyzing the impact of mass shootings focuses on public mass shootings (15, 19, 20, 24) because they typically involve higher victim counts and generate more media attention (35) than other types of gun violence involving three or more victims (e.g. familicide or gang-related shootings). We conducted an extensive search across multiple databases on mass shooting events to identify the subset occurring in schools and college campuses, workplaces, religious institutions, recreational areas and festivals, shopping venues, and other public settings. The list of 44 public mass shooting we identify is comprehensive, including the deadliest high-profile shootings during the decade under study (e.g. Las Vegas Harvest Music Festival, Orlando Florida Pulse Night Club, and Sandy Hook Elementary), as well shootings generating considerably less national media attention (e.g. Marysville Pilchuck High School Shooting, Carson City IHOP shooting, and Don Carter Lanes).

Importantly, public mass shootings sometimes occur in event chains or temporal clusters, whereby one or more shooting occurs shortly in time after an initial event. This phenomenon is sometimes referred to by journalists and scholars as a “contagion” or “copy cat” effect (62). Several of the public mass shootings on our list occurred within several days of another shooting, such as the Gilroy Garlic Festival, El Paso Walmart, and Dayton Ned Pepper’s Bar shootings in 2019. Within our data, 37 shootings can be analyzed as standalone events, whereas 7 shootings, given their proximity to others, are analyzed in event clusters. Our analysis includes 3 event clusters: the 2016 Kalamazoo County + Hesston Excel Industries cluster; the 2019 Gilroy + El Paso + Dayton cluster; and the 2021 Atlanta Massage Parlors + Boulder CO King Soopers supermarket cluster.^i^ In total, these 40 public mass shooting events (37 standalone shootings, 3 shooting clusters) span a decade of time and are diverse in terms of geographic region, setting of shooting, and characteristics of the shooter and victims. We include a map (Fig. A1) and detailed table of the shootings (Table A1).

Figure 1 displays the cumulative proportion of media coverage in the nation’s top-50 news sources (as categorized by Media Cloud) that mentioned the terms “mass shooting” for the week following each shooting. The plot is sorted by highest to lowest coverage and grouped into quartiles. Existing scholarship addresses the question of why some public mass shootings garner more media attention than others (35). Public mass shootings, like any event occurring in a specific place and time, are unobserved by citizens outside of the immediate vicinity of the shooting or the confines of local social and media networks. As such, national media attention serves as a key vehicle for raising widespread public awareness of a shooting event, and thus, in bringing about a change in behavior across the populace. With this in mind, we present results for *high-publicity* and *low-publicity* public mass shootings, which we distinguish as those receiving above vs. below median levels of national media attention. However, to keep the presentation of results as succinct as possible, our figures focus on displaying the estimated effects of high-publicity public mass shootings, which we define as those receiving above median levels of national media attention (the “Top 20” shooting events in Fig. 1). All results presented below focus on this subset of high-publicity shooting events and we present results for low-publicity public mass shootings (i.e. below median or “Bottom 20” shooting events in Fig. 1 and Figs. A3 to A7)  in the Appendix. This said, to aid readers in drawing comparisons in effects by level of media attention, our figures include meta-analytic estimates summarizing average effects across outcomes for shooting events receiving the *most* media attention (“Top 10”), all high-publicity shooting events receiving above median media attention (“Top 20”) and low-publicity shooting events (“Bottom 20”).

## Results

### Information seeking

**Fig. 2.**
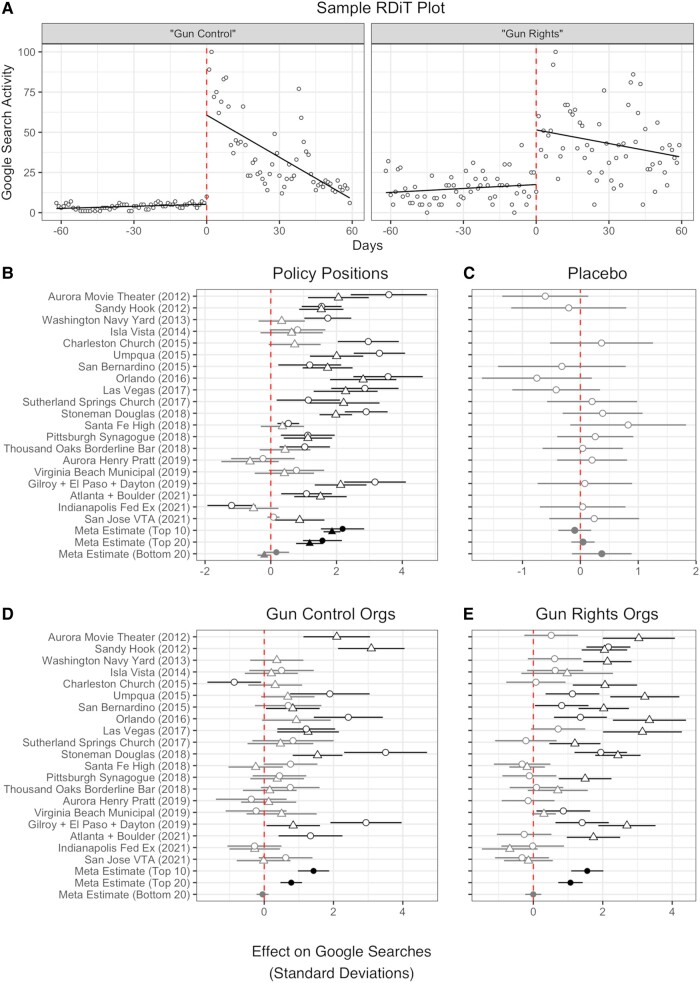
Effect of public mass shootings on internet search behavior. A) Displays daily Google Trends search data for “Gun Control” before and after the Stoneman Douglas shooting. The remainder of the plots display RDiT treatment effect estimates with 95% CIs. B) Circles indicate “Gun Control” and triangles “Gun Rights” searches. C) Estimates are for “recycling” searches. D) Circles indicate “Everytown for Gun Safety” and triangles “Brady Campaign” searches. E) Circles indicate “Gun Owners of America” and triangles “NRA” searches. For the three bottom rows of B–E) “Top 10,” “Top 20,” and “Bottom 20” indicate meta-analytic estimates for shootings by rank of media coverage received. Point estimates that are colored gray in Panels B–E indicate that the CIs includes 0; point estimates in black are statistically significant. Missing estimates arise when there is no overlap between the time series outcome variable we measure and when a shooting occurred or insufficient data to estimate an effect. B–E) The units are changes in SDs.

Figure 2 displays the effect of public mass shootings on Americans’ information-seeking behavior on gun policy. We begin by presenting a plot of the raw data of Google searches following one example event—the 2018 Stoneman Douglas High School shooting—to illustrate what a sample case looks like, before presenting RDiT coefficient plot estimates across all of our other outcomes and shootings. The plots in panel A present raw daily Google search data for “Gun Control” and “Gun Rights,” which are rescaled to range between 0 and 100, together with a best-fit linear regression on both sides of the event cutpoint, for the two months before and after the Stoneman Douglas shooting. This figure illustrates the clear and substantial jump in internet search interest of 65 points (2.26 SDs) for “Gun Control” and 46 points (2.27 SDs) for “Gun Rights” following the shooting. These effects are *large*—being both statistically and substantively significant by any reasonable benchmark—and are indicative of a meaningful increase that lasts long after the shootings.

The plots in the middle of Fig. 2 display the RDiT estimates scaled by the SD of each outcome for each of the mass public shooting events we analyze on Google searches for gun policy positions (panel B), a “placebo” search term (“recycling”) presumably unrelated to gun violence or mass shootings (panel C), and gun policy organizations (panels D and E). panel B examines the effect of public mass shootings on internet search interest in gun control (circles) and gun rights (triangles). Because publicly available Google Trends data is rescaled depending on the date range of data collected, it is not possible to know the actual number of raw searches on any given day, so we rely on SD shifts to characterize the magnitude of the effect size (*d*) in political activity. As a benchmark for interpreting substantive significance, we note that many scholars have used the heuristic of three cutoffs that demarcated small (d=0.2), medium (d=0.5), and large (d=0.8) effect sizes from one another (63). Each analyzed mass shooting spanning from Sandy Hook in 2012 to Atlanta + Boulder in 2021 caused statistically significant and substantively large surges, from between −1.19 (“Gun Control” searches following Indianapolis Fed Ex [2021]) and 3.59 (“Gun Control” searches following Aurora Movie Theater [2012]) SD shifts.

We also present meta-analytic estimates of treatment effects in the final three rows of the coefficient plots in Fig. 2. We present meta estimates for the 10 shooting events listed at the top of Fig. 1 with the most media coverage “(Top 10),” the 20 high-publicity shooting events with above median levels of media coverage “(Top 20),” and the 20 low-publicity shooting events that garnered the least (i.e. below median) media coverage “(Bottom 20).”^j^ We include full plots of RDiT estimates for these shootings in the Appendix. Across the 10 shooting events with the most media coverage, we find an average increase in “Gun Control” searches of 2.18 SDs and “Gun Rights” searches of 1.86 SDs. Meta-analytic estimates for the top 20 shootings are two-thirds to three-quarters as large as those of the top 10. And, as expected, estimates for the bottom 20 low-publicity shooting events are a relatively precise 0. This highlights the expected moderating role that the media play in the effects we document. Again, the effects for public mass shootings with high media coverage are *large*—showing a massive increase in gun-related information seeking following shootings. In stark contrast, panel C displays null RDiT estimates for the impact of public mass shootings on an unrelated search terms (“Recycling”). These null results confer validity to the findings presented in panel B by illustrating that mass public shootings do not cause immediate shifts in public interest in an issue unrelated to gun violence or policy.

Accompanying searches for gun policy positions, panels D and E demonstrate that, following high-publicity public mass shootings, Americans go beyond seeking information about gun policy positions by engaging in internet searches for leading gun control and gun rights advocacy organizations, such as Everytown for Gun Safety (panel D, circles), the Brady Campaign to Prevent Gun Violence (panel D, triangles), Gun Owners of America (panel E, circles), and the NRA (panel E, triangles). We find −0.87 (searches for “Everytown for Gun Safety” following Charleston Church Shooting) to 3.5 SD (searches for “Everytown for Gun Safety” following Stoneman Douglas) jumps in search activity for gun policy organizations, an average of 1.42 to 1.55 SDs increases (Top 10) for gun control and gun rights organizations, respectively. By any standard, these effects are large.

**Fig. 3.**
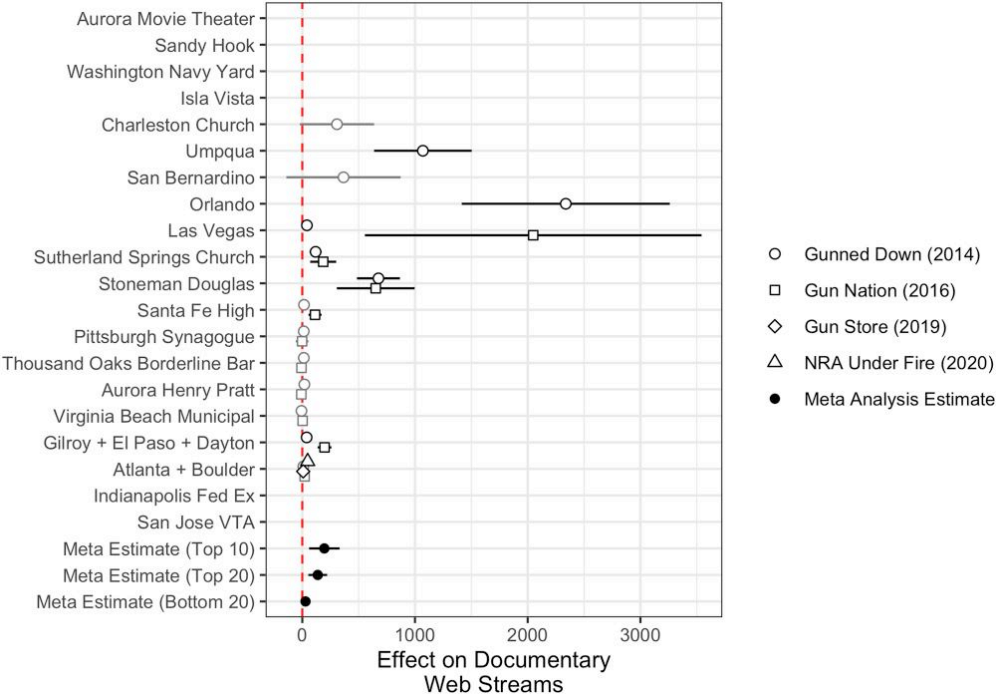
Effect of public mass shootings on documentary streams. RDiT point estimates with 95% CIs. Gray point estimates indicate that the CIs includes 0; black point estimates are statistically significant. Missing estimates arise when there is no overlap between the time series outcome variable we measure and when a shooting occurred or insufficient data to estimate an effect. For the three bottom rows of the figure, “Top 10,” “Top 20,” and “Bottom 20” indicate meta-analytic estimates for shootings by rank of media coverage received.

Figure 3 displays the RDiT estimates for the impact of public mass shootings on an alternative indicator of information-seeking: watching a documentary about gun violence and firearms regulations. For the PBS documentary *Gunned Down*, which aired in 2014, we see that all but two of the high-publicity public mass shootings that occurred after the airing of the documentary caused a significant spike in streaming activity on their web interface. These effects, reported in terms of number of raw streams of the documentary, are relatively smaller, ranging from 0 additional views (*Gunned Down* following Virginia Beach Municipal) to 2,337 additional views (*Gunned Down* following Orlando) relative to a median daily stream baseline of 43. We find a similar range of spikes in web streams for *Gun Nation*, which range from 0 additional streams following the Aurora Henry Pratt shooting to 2,049 additional streams following the Las Vegas shooting. The effect of the Atlanta and Boulder shooting on streams of *NRA Under Fire*, a documentary released in March 2020, is statistically significant but relatively small, at 47 additional streams, relative to a median daily stream baseline of 41. Last, we observe a small but statistically significant jump of 8 additional streams of *Gun Store* on YouTube following the Atlanta + Boulder shootings, relative to virtually no streams (median value of 0) leading up to that shooting. While these effects may seem inconsequentially small (a meta-analytic effect of 196 streams on average), three caveats are due: first, the meta-analytic effect relative to the base rate is large; second, streaming an educational documentary on an internet-connected device is a time-consuming, and thus “high cost,” activity relative to less costly activities like Google searches; third, we highlight that we have data only on certain web streams on proprietary digital streaming platforms or on YouTube—these metrics do not capture the consumption of the documentaries when they are aired on live television or on different platforms like Amazon Prime or *Vimeo*.

It is important to note that internet searches *cannot* be equated with political preference or endorsement, as users in favor of gun control could search for information about gun rights (i.e. counter-attitudinal searches). This said, the findings in Figs. 2 and 3 provide support for one firm conclusion: high-publicity public mass shootings over the past decade caused clear, consistent, and sizable surges in political information-seeking among the American public. This information-seeking activity encompasses multiple behaviors (searching the internet and streaming documentaries) and targets different types of political information (policy positions and advocacy organizations). Critically, we find that this information-seeking activity is not limited to one side of the policy debate on firearms in America. These figures also reveal that shootings garnering scant media attention (i.e. “Bottom 20”) on average had little-to-no effect on these information seeking behaviors.

### Political discussion on twitter

**Fig. 4.**
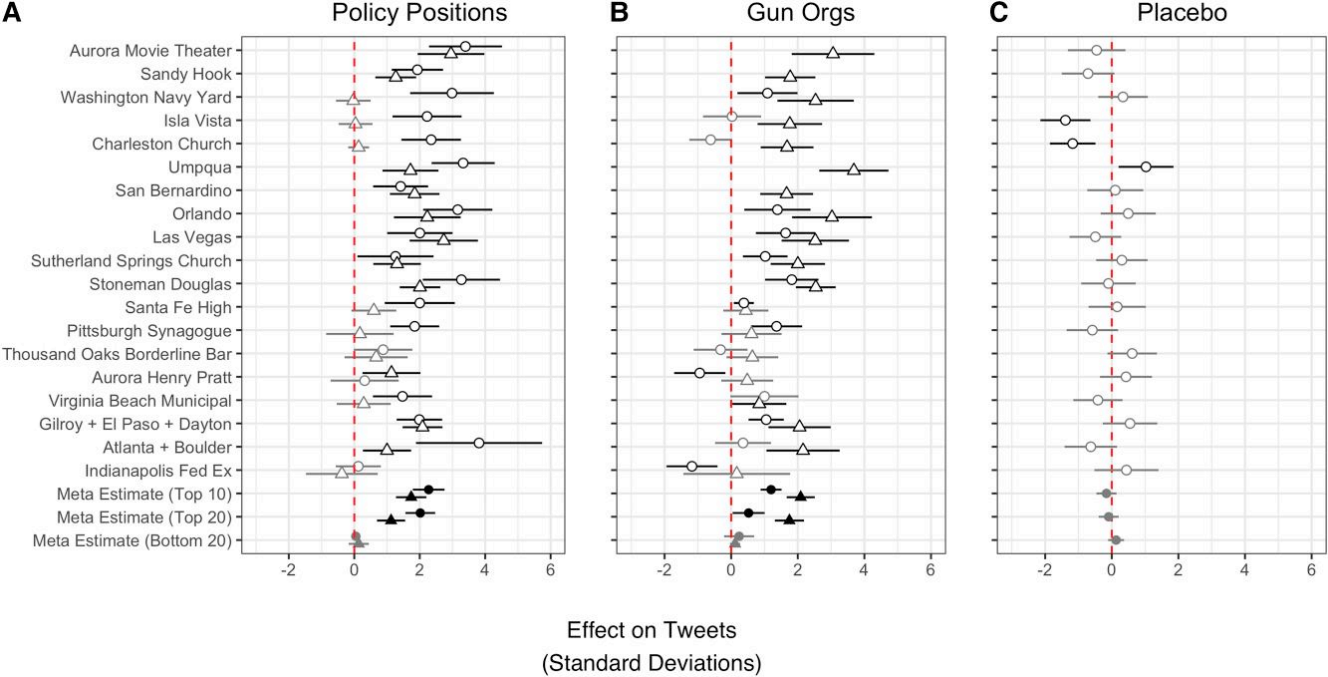
Effect of public mass shootings on social media discussion. RDiT treatment effect estimates with 95% CIs. A) Circles indicate effects on “#guncontrol” and triangles on “#gunrights” tweets. B) Circles indicate effects on “#everytown” and triangles “#NRA” tweets. C) The placebo outcome is tweets including “#recycling.” For the three bottom rows of panels A–C, “Top 10,” “Top 20,” and “Bottom 20” indicate meta-analytic estimates for shootings by rank of media coverage received. Gray point estimates indicate that the CIs includes 0; black point estimates are statistically significant. Missing points arise when there is no overlap between the time series we measure and when the shootings occurred or we have insufficient data to estimate an effect.

Figure 4 presents the results from our analysis of discourse about guns on Twitter. We present RDiT estimates for the effect of public mass shootings on Tweets using hashtags mentioning policy positions (“#guncontrol” and “#gunrights,” circles and triangles in panel A), Tweets using gun policy advocacy organization hashtags (“#everytown” and “#NRA,” circles and triangles in panel B), and Tweets with a hashtag unrelated to firearms or gun policy (“#recycling,” panel C).

We consistently find that high-publicity public mass shootings cause large spikes in gun-related policy conversation mentioning “#guncontrol” or “#gunrights.” The effects range from null to 3.81 SDs (“#guncontrol” following Atlanta + Boulder). Our meta-analytic estimate of the effects for the top-10 shootings is between 1.73 and 2.27 SD increases for “#guncontrol” and “#gunrights” tweets, respectively, or between n=4,875 and 6,397 additional gun policy-related Tweets, on average, immediately following a high-media attention shooting. Prominent public mass shootings also lead to large spikes in Twitter comments that mention specific gun policy organizations, with slightly smaller effects ranging from null up to 3.68 SDs, an average spike of between 1.20 and 2.09 SD or an additional n=3,381 to 5,889 Tweets. Importantly, we observe almost exclusively null results (e.g. a relatively precise null meta-analytic effect of −0.16 for top-10 shootings) in panel C for a Twitter topic that is unrelated to gun policy (“#recycling”). Finally, the bottom rows of Fig. 4 reveal that low-publicity public mass shootings had no detectable effect on discussion of gun policy on Twitter.

In sum, the results in Fig. 4 provide evidence that high-publicity public mass shootings over the past decade led to consistent and sizable spikes in social media discussion of gun policy positions and advocacy organizations. Given that this analysis is drawn from the entire universe of Tweets during the time period under study, it is important to note that these findings are comprehensive. However, while we have evidence that many of the Tweets mentioning “#guncontrol” or “#everytown” are accompanied by language calling for strengthening gun laws and Tweets mentioning “#gunrights” or “#NRA” are accompanied by language calling for gun rights (see Fig. A2 for examples), there are a nonnegligible number of Tweets that include both or express mixed sentiments. As a result, while these results are suggestive of increases in discussion on both sides of the policy debate, the firmest conclusion that can be drawn is that the American public consistently responds to high-profile public mass shootings with heightened online discussion of gun policy positions and advocacy organizations.

### Purchases of political flags

**Fig. 5.**
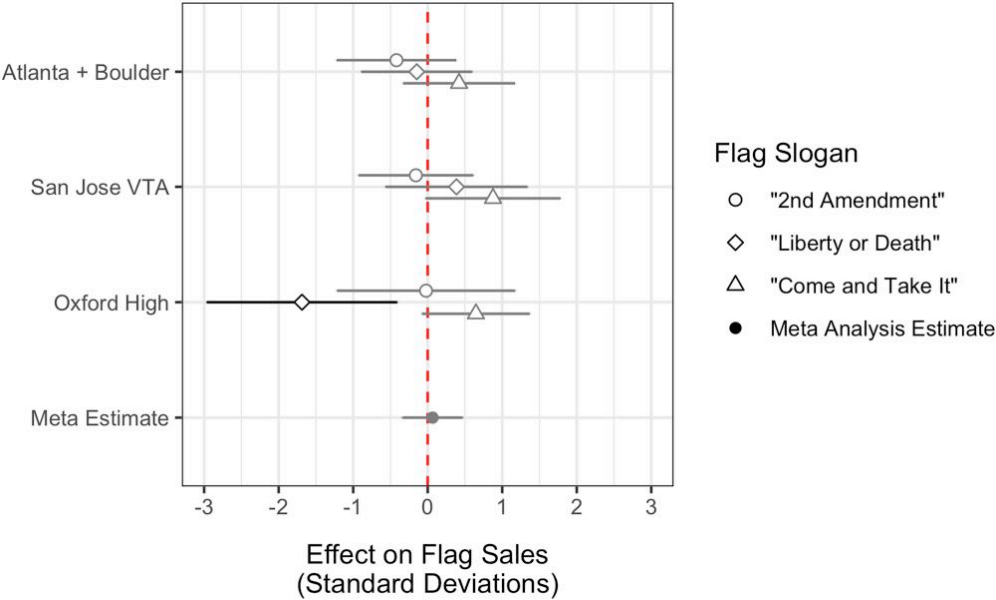
Gun rights flag sales. RDiT treatment effect estimates with 95% CIs for flag sales of three different pro-gun flags. Gray point estimates indicate that the CIs includes 0; black point estimates are statistically significant.

This section investigates whether or not our findings for Twitter discourse extend to other means of publicly expressing one’s views on guns, such as purchasing political flags for display. Our data on flag sales cover the time period when three public mass shootings occurred—two higher publicity events (Gilroy + El Paso + Dayton and San Jose VTA) and one lower publicity event (Oxford High). As shown in Fig. 5, we find that none of these events lead to a statistically significant increase in flag purchases. The meta-analytic estimate across flags and shootings is statistically indiscernible from zero. In sum, when analyzing an outcome seemingly unique to the gun rights side of the debate—purchasing political flags for display with gun rights messages—we fail to uncover evidence that recent public mass shootings lead those presumably in favor of gun rights to purchase pro-guns flags as means of expressing their views. To be clear, over 46,000 of these three popular gun rights flags were sold by ANLEY alone between January 2021 and January 2022, indicating that this is an activity gun rights supporters engage in. Our findings here simply suggest it may not be an act they engage in *more* in the wake of public mass shootings.

### Petition signing and donations

**Fig. 6.**
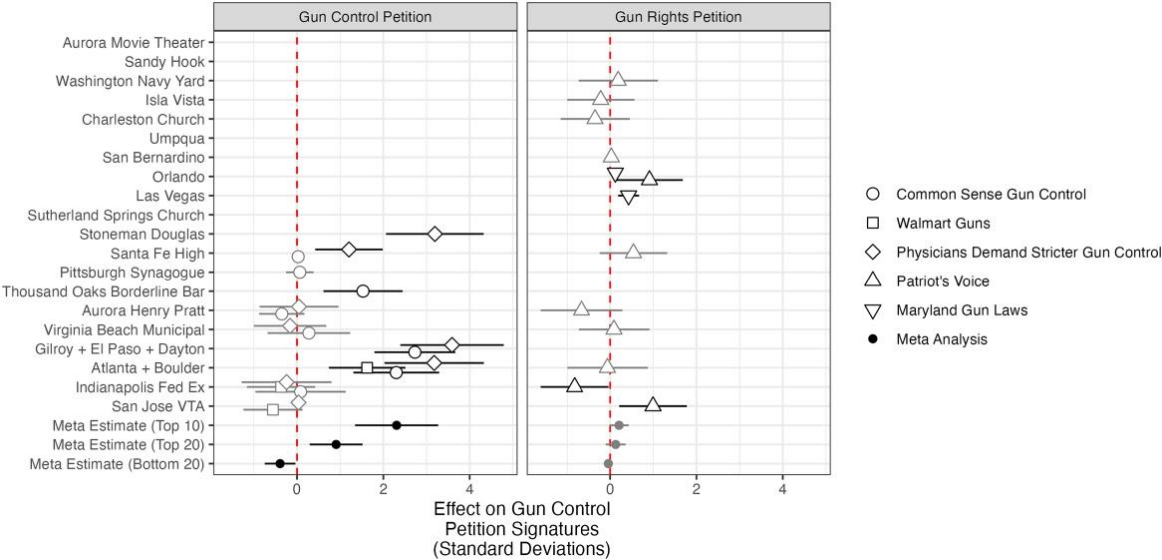
The effect of public mass shootings on petition signing. RDiT treatment effect estimates with 95% CIs. Gray point estimates indicate that the CIs includes 0; black point estimates are statistically significant. Missing points arise when there is no/insufficient overlap between the time series we measure and when the shootings occurred. For the three bottom rows of the figure, “Top 10,” “Top 20,” and “Bottom 20” indicate meta-analytic estimates for shootings by rank of media coverage received.

We now turn to the more unequivocally partisan political acts of signing a gun policy petition and donating to a gun policy PAC. Figure 6 displays the RDiT results for our analysis of petition signatures for major gun control and gun rights petitions. For the shooting events for which we have data on gun control petitions, we observe some very large spikes in signatures following shooting events: these spikes range between 0 and 3.59 SDs (following Gilroy + El Paso + Dayton). On the gun control petition side, the meta-analytic estimates in the bottom rows make it clear that signatures to prominent Change.org petitions significantly spiked following high-publicity shooting events (e.g. Stoneman Douglas) but experienced little change following low-publicity shooting events (e.g. Aurora Pratt). Turning to gun rights petitions in the right-side graph, we find inconsistent and mostly null effects of public mass shootings on signature activity. For example, signatures to “Patriot’s Voice” significantly increased after the Orlando nightclub and San Jose VTA shootings but significantly decreased following the Indianapolis FedEx shooting. The meta-analytic estimates in the bottom row indicate substantively small and statistically insignificant effects, on average, of high- and low-publicity shootings on signatures to these gun rights petitions.

**Fig. 7.**
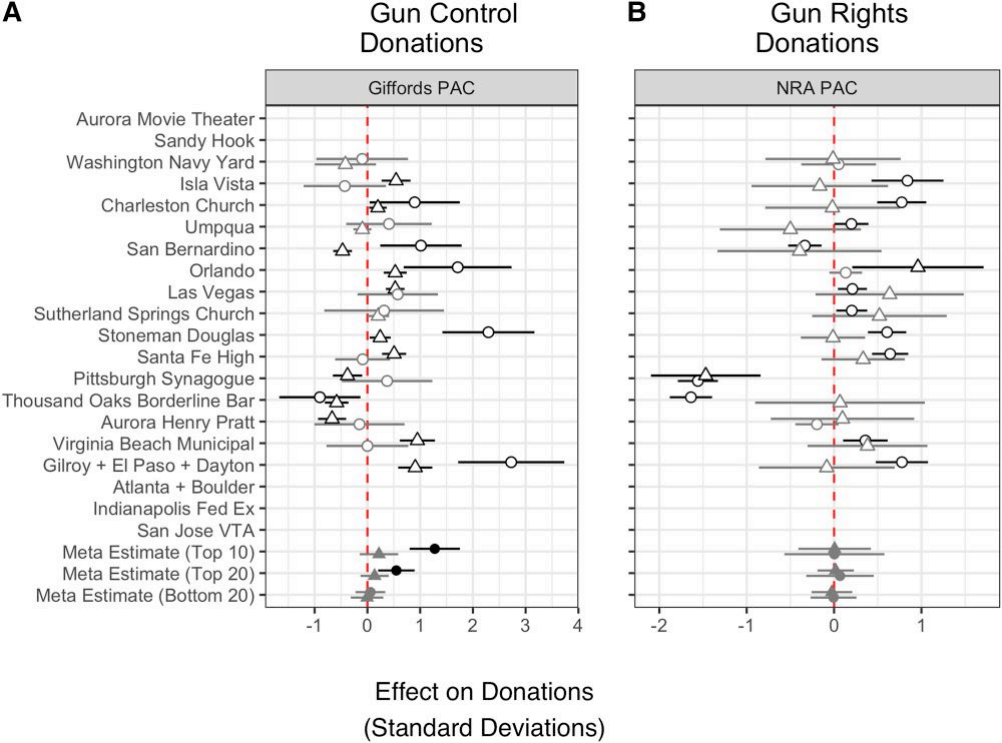
The effect of public mass shootings on donations. RDiT treatment effect estimates with 95% CIs. A and B) Triangles are estimates for number of donations and circles for donation amounts. For the three bottom rows of panels A and B, “Top 10,” “Top 20,” and “Bottom 20” indicate meta-analytic estimates for shootings by rank of media coverage received. Gray point estimates indicate that the CIs includes 0; black point estimates are statistically significant. Missing points arise when there is no/insufficient overlap between the time series we measure (i.e. the dates the organization was registered with the FEC) and when the shootings occurred.

In Fig. 7, we display the RDiT treatment effect estimates for all relevant shootings on SD shifts numbers of donation (triangles) and dollar amounts (circles) to the Giffords PAC and the NRA PAC. Contrary to previous analyses, we find far less conclusive evidence that public mass shootings spur donations to gun policy-related advocacy organization PACs. With respect to the Giffords PAC, we observe positive surges in donation amounts in 5 of 15 shootings and in the number of donors in 7 of 15 shootings, though the positive effects for number of donations are much smaller in magnitude. Our meta-analysis of the highest profile shootings suggests surges, on average, in donation amounts but not in number of donations to Giffords PAC. Effects are also mixed for the NRA PAC donations. While we observe positive spikes in the number of donors in 9 of 15 shootings, they are small in magnitude, and we only find an increase in dollar amounts following 1 of the 15 shootings. On average, effects for numbers and amount of donations to NRA PAC are precisely null. In order to directly compare the weight of donations to gun control vs. gun rights PACs, we also calculate the difference in number of raw donations to NRA PAC compared to Giffords PAC (#Giffords–#NRA) and again estimate treatment effects following each of the mass shootings for which we have data. In 8 of the 15 shootings, the RDiT estimates are indistinguishable from zero, while the Thousand Oaks Borderline Bar, San Bernardino, and Pittsburgh Synagogue shootings generated larger surges for Giffords PAC (between 0.26 and 1.33 SDs relative increases) and the Stoneman Douglas, Santa Fe High, Isla Vista, and Charleston Church shootings for the NRA PAC (0.47 to 0.59 SD relative increases).

### The average and countervailing effects on political activity

Is there an overall political tilt in the activity generated by public mass shootings? That is, when looking across shootings and outcomes, do we see roughly equivalent effects or do we observe greater effects for activity on a given side of the gun policy debate? To answer this question, we estimated meta-analytic effects using RDiT estimates of SD shifts following all shootings for which we had data for Google searches for “gun control,” “gun rights,” gun control organizations (“Brady Campaign” and “Everytown for Gun Safety”), and gun rights organizations (“Gun Owners of America” and “NRA”), all “#guncontrol,” “#gunrights,” “#everytown,” and “#NRA” Tweets, all gun rights and gun control petitions, and then all donations (both dollar amounts and volume) to gun rights (NRA) and gun control (Giffords) PACs. We estimate the meta-analytic effects for the 10 shootings with the most media coverage “Top 10” (panel A), the top 20 shootings with above median levels of media coverage “Top 20” (panel B), and the 20 shootings that garnered the least media coverage “Bottom 20” (panel C).

**Fig. 8.**
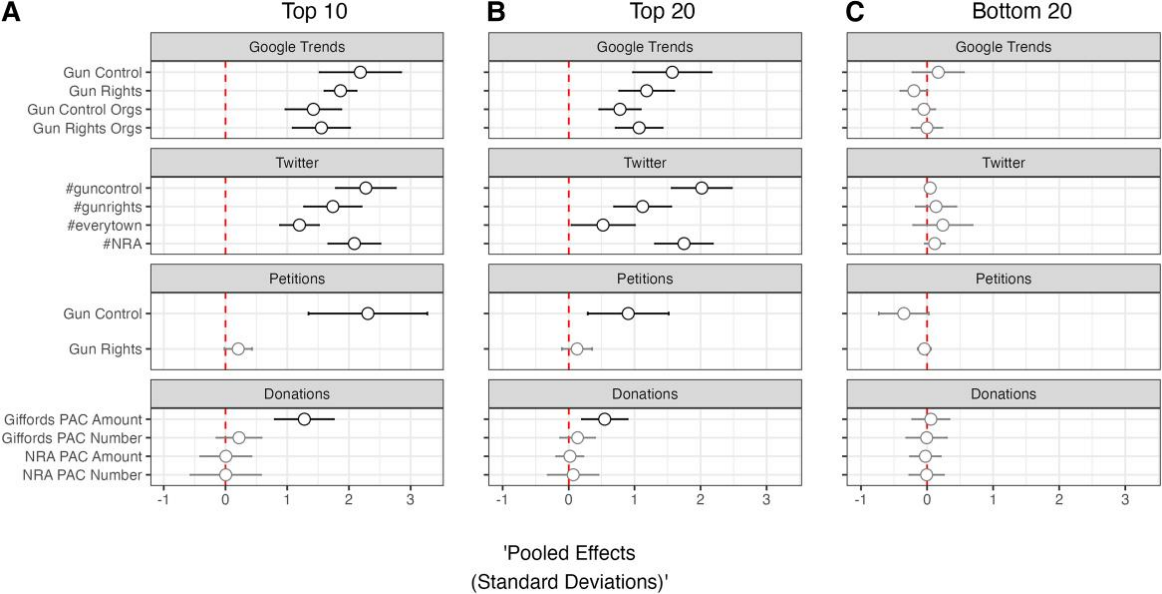
Meta-analysis of effects across outcomes by national media attention. Pooled RDiT treatment effect estimates with 95% CIs. Gray point estimates indicate that the CI includes 0; black point estimates are statistically significant. “Top 10” (Panel A), “Top 20” (Panel B), and “Bottom 20” (Panel C) refer to rank of shooting based of media coverage received.

The results from this analysis are presented in panels A, B, and C in Fig. 8. Looking at just the top-10 shootings in panel A, we observe large changes in internet search and social media activity on both the “gun control” and “gun rights” sides of the equation. For petition signing, which are more unequivocally partisan, on average we observe large changes in signing of gun control petitions but little change in signing of gun rights petitions. Finally, with respect to donations to the PACs of gun policy advocacy organizations, we find that the average shooting in our sample elicits larger sized donations to a gun control organization but not a greater number of donations. These latter finding makes sense given that income constrains donation behavior and there is little reason to expect that public mass shootings trigger broad changes in disposable income. As such, it stands to reason that shootings appear to encourage those already able to give to donate more rather than expanding the number of donors in the gun policy arena. We fail to observe changes in the number or amount of donations for the average shooting in our sample for the gun rights organization we consider. Moving to panel B, the “Top 20” shootings, we find a similar pattern of effects with some minor differences: first, when focusing on the 20 shooting events receiving above median news coverage, we observe a more pronounced pattern of shootings on average generating more tweets mentioning gun control than gun rights and generating more tweets mentioning the NRA than Everytown; second, average effects on petition signing and donations are smaller in magnitude. Concluding with panel C, the 20 shootings that garnered far less media coverage, we find null effects across all outcomes.

One might wonder whether the weaker effect on average of high-publicity public mass shootings on gun rights Tweets, petition signatures or donations derive from these activities being more a part of the political participation “repertoire” of liberals who favor gun control compared to conservatives who tend to oppose it. In other words, do these tests stack the deck by choosing acts that those in support of gun rights simply do not engage in? Survey data suggest against this, with several representative samples of American adults finding relatively small differentials between those favoring gun rights vs. gun control in self-reported rates of expression of opinions about gun policy on Twitter or signing of petitions on gun policy (11). Moreover, the same surveys find that those supporting gun rights are *more likely* than those supporting gun control to report donating money to a gun policy organization. Such findings render it unlikely that the differences uncovered in Fig. 8 derive from underlying asymmetry in the types of acts engaged in by supporters vs. opponents of gun control.

### Heterogeneity by proximity and race/ethnicity of victims

Previous research argues that the effect of public mass shootings may be conditioned by proximity to the location of shootings, e.g. (20) and the race/ethnicity of shooting victims (65). These works suggest that public engagement with gun policy will be greater among those residing closer to public mass shootings and when the victims of a shooting are mostly white. Appendix B presents the results from analyses exploring the effect of the shootings in our data by proximity using in-state vs. out-of-state as the measure of proximity (Fig. A8) and by the the percentage of shooting victims that were estimated by to be non-Latino white (Fig. A11). The results in Appendix B reveal little discernible heterogeneity across these dimensions. First, for the outcomes in our data where geocodes were readily available (e.g. petitions and donations), we observe similar effects of shootings on engagement arising from the state where shootings occurred (i.e. “in-state”) compared engagement in all other states (i.e. “out-of-state”). Second, after retrieving a list of the full names of all victims for each shooting event in our data and estimating the race/ethnicity of each victim (procedure described in Appendix B), we were able to generate an estimate for each shooting event of the percentage of victims that were non-Latino white. As a quick aside, the amount of media coverage received by a shooting event is not correlated with the ethno-racial composition of its victims (Fig. A10); rather, media coverage is heavily positively correlated with the number of victims, which comports with previous research (35). We reestimated RDiTs by tercile of % non-Latino white of victims and find little evidence that public engagement with gun policy systematically varies by race-of-victim.

## Conclusion

This study demonstrates that high-publicity public mass shootings cause large surges in myriad forms of public engagement with gun policy. These findings belie the standing wisdom that ordinary Americans do not engage in action oriented toward gun control following the occurrence of public mass shootings. Critically, our analysis finds that these upswings in engagement are countervailing: in many instances, we observe surges in activity on both sides of the policy debate; however, when averaging across shootings and outcome measures, contrary to popular claims about an “enthusiasm gap,” we find the shootings-induced activity tilts toward gun control. Finally, our analysis reiterates the vital role of the media in shedding light on these tragic events, as shootings receiving relatively little media attention, on average, did not instigate public engagement with gun policy. In contrast, shootings receiving extensive media coverage generate large effects. If most Americans do not learn about a public mass shooting, there is little chance the shooting will spur widespread political activity. Following the public mass shooting at the Old National Bank in Louisville, KY, in 2023 April 10, a letter to the editor of the *Los Angeles Times* complained “we are becoming so inured to mass shootings that the one in Louisville made only page A-12 of the following day’s print LA Times. If we want lawmakers to do something about this problem, we cannot bury the stories in the back of the paper as if we don’t care.”^k^

While this article offers the most extensive analysis of public engagement with gun policy following public mass shootings to date, we see several directions for future research. First, future scholarship could analyze additional types of engagement, such as attending a march or rally or contacting an elected official. Second, publicly available FEC data on donations to major gun policy PACs is quite limited; thus, future research could build on our analysis of PAC donations by striving to obtain data on small donations made to a wider range of prominent gun policy organizations. Third, while our analysis focuses on public mass shootings that occurred over the past decade (2011 to 2021), future research could extend our analysis to shootings occurring prior to 2011 or those occurring over the past few years. Fourth, while prior work finds little direct effect of public mass shootings on electoral behavior (24, 25), future research could explore whether the surges we observe in nonelectoral engagement with gun policy have downstream effects on electoral behavior. In other words, an open question for future research is whether public mass shootings indirectly heighten voter turnout or Democratic vote choice by first elevating forms of nonelectoral engagement (e.g. information seeking, social media discussion, petition signing, or donating money), which subsequently alter turnout and vote choice. Fifth, while we find little evidence that geographic proximity to shootings or the race/ethnicity of victims condition their effects, future research could explore other possible moderators, such as the amount of prior exposure to gun violence or having school-aged children. Finally, scholars would do well to compare the downstream effects of increases in various types of public engagement with gun policy on the beliefs and actions of elected officials.

## Supplementary Material

pgad407_Supplementary_Data

## Data Availability

The data underlying this article are available at the Harvard Dataverse: https://dataverse.harvard.edu/dataset.xhtml?persistentId=doi:10.7910/DVN/TTZGFV

## Notes

a This documentary is available at *The Guardian’s* website (link).

b
https://financesonline.com/twitter-statistics/

c Pew Research Center’s American Trends Panel Poll, Question 24, 31118576.00025, Ipsos, (Cornell University, Ithaca, NY: Roper Center for Public Opinion Research).

d Survey Center on American Life, 2020 American National Social Network Survey, Question 26, National Opinion Research Center and Pew Research Center for the People & the Press, 2020 American Trends Panel Wave 78, Question 4.

e We attempted to collect data for several other popular gun control and gun rights petitions on Change.org but were unable to retrieve contact information for the petition’s creator. Change.org makes the name of petition creators public but does not provide accompanying contact information. Moreover, support staff at Change.org would not contact petition creators on our behalf. The data we obtained were due to locating contact information for petition creators through internet searches using their name.

f We do not include analyses for The Brady Campaign to Prevent Gun Violence PAC, which stopped raising and spending money around 2010, or Everytown for Gun Safety Action Fund PAC, which was established relatively recently and has not yet raised substantial amounts of money.

g Some scholars have noted that RDiTs are conceptually similar to interrupted time series (61).

h
https://www.gunviolencearchive.org/methodology

i For these event chains, we treat the day of the last shooting in the chain (Kalamazoo, Dayton, and Boulder, respectively) as the “treatment” date. This decision yields RDiT estimates that are likely biased downward given the spike in activity in the “control” period that may have been activated by the previous shootings, so we consider any treatment effects for these shootings to be conservative estimates of the effect of these shootings on each outcome.

j Generic inverse variance meta-analyses of RDiT effects (64) were estimated via random effects regression using the meta package in R.

k
https://www.latimes.com/opinion/letters-to-the-editor/story/2023-04-12/run-hide-fight-louisville-mass-shootings

## Previous Presentation

These results were not previously presented.

## Preprints

No preprint of this article has been published.
