# Supplementary material for: Public mass shootings cause large surges in Americans’ engagement with gun policy
Source: PNAS Nexus. 2023 Nov 29;2(12):pgad407. doi: 10.1093/pnasnexus/pgad407 (PMC10726999; doi:10.1093/pnasnexus/pgad407)
Supplement: pgad407_Supplementary_Data [file pgad407_supplementary_data.pdf]

Supplemental Information for: "Public mass shootings cause large surges in Americans' engagement with gun policy"

Contents

1 Appendix A **2**
1.1 Regression Discontinuity in Time (RDiT) . . . 2
1.2 Details on Data . . . 3
1.3 Sample Tweets . . . 5
1.4 Lower Media Attention Shootings . . . 6

2 Appendix B **11**
2.1 Heterogeneous Treatment Effects By Proximity . . . 11
2.2 Heterogeneous Treatment Effects By Race of Victim . . . 12

1 Appendix A

1.1 Regression Discontinuity in Time (RDiT)

The as-good-as random timing of these public mass shootings assuage concerns about "anticipation effects" present with other RDiT designs focusing on planned interventions like the enactment of different policies (Hausman and Rapson, 2018), as public mass shootings are never publicly planned nor anticipated. Regression discontinuity designs (RDD) leverage as-if-random variation around these arbitrary cutoffs to estimate local causal effects that correspond well to certain RCT treatment effects (Wing and Cook, 2013). The "running variable" we use is time—the number of days before (which take negative values) and after (which take positive values) the date of each public mass shooting.

We set the cutpoint (where the running variable, c=0) to the day of each public mass shooting and trim the pre-treatment and post-treatment data to 2 months before and after each event, though the ultimate bandwidth used to estimate the regression is selected for each model using the Imbens-Kalyanaraman optimal bandwidth Imbens and Kalyanaraman (2012) estimated with the `rddtools` package implementation in R (Stigler and Quast, 2016). Given that we have high-frequency data (daily), this yields sufficient sample on either side of the discontinuity to estimate local regressions while minimizing the interference of other events that may occur before or after any given event. We model the running variable using a parametric polynomial regression.

Scholars have noted the similarity between the RDiT approach and those of a (high-frequency) interrupted time series or event study design (Hausman and Rapson, 2018). We use the regression discontinuity terminology given that our running variable is high frequency, being measured daily, but acknowledge the conceptual overlaps between these similar methods.

1.2 Details on Data

Figure A1: Geographic Distribution of Shootings

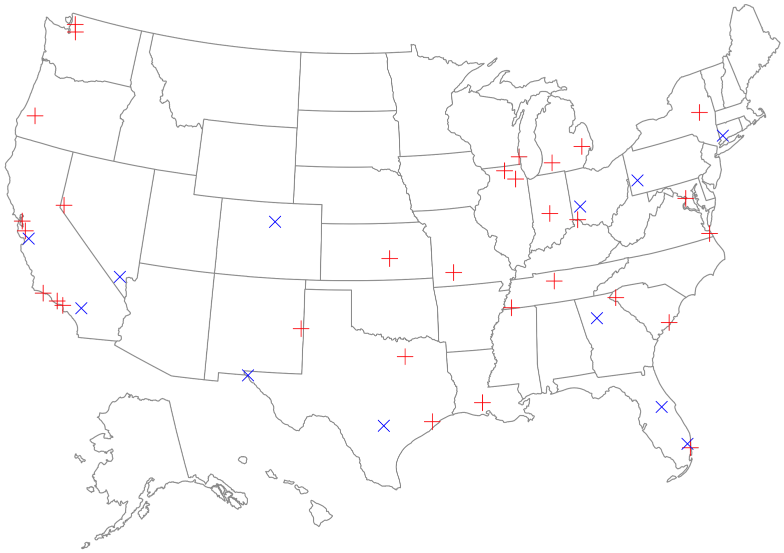

Note: Blue X are shootings that obtained highest levels of media coverage and red + are remaining lower-media shootings.

Table A1: Public Mass Shootings Used

Mass Public Shooting	Date	Location	Deaths	Injuries
Carson City IHOP	September 6, 2011	Carson City, NV	5	7
Oikos University	April 2, 2012	Oakland, CA	7	3
Aurora Movie Theater	July 20, 2012	Aurora, CO	12	70
Wisconsin Sikh Temple	August 5, 2012	Oak Creek, WI	8	3
Sandy Hook School	December 14, 2012	Newtown, CT	28	2
Herkimer County	March 13, 2013	Herkimer County, NY	5	2
Santa Monica College	June 7, 2013	Santa Monica, CA	6	4
Washington Navy Yard	September 16, 2013	Washington DC	13	8
Isla Vista	May 23, 2014	Isla Vista, CA	7	14
Marysville Pilchuck High School	October 24, 2014	Marysville, WA	5	3
Charleston Church	June 17, 2015	Charleston, SC	9	1
Lafayette Movie Theater	July 23, 2015	Lafayette, LA	3	9
Umpqua Community College	October 1, 2015	Roseburg, OR	10	8
San Bernardino Holiday Party	December 2, 2015	San Bernardino, CA	16	24
Kalamazoo	February 20, 2016	Kalamazoo County, MI	6	2
Hesston Excel Industries	February 25, 2016	Hesston, KS	4	14
Orlando Pulse Nightclub	June 12, 2016	Orlando, FL	50	58
Cascade Mall	September 23, 2016	Burlington, WA	6	0
Townville Elementary	September 28, 2016	Townville, SC	2	3
Fort Lauderdale Airport	January 6, 2017	Broward County, FL	5	42
Clovis Library	August 28, 2017	Clovis, NM	2	4
Las Vegas Music Festival	October 1, 2017	Las Vegas, NV	61	867
Sutherland Springs Church	November 5, 2017	Sutherland Springs, TX	27	22
Stoneman Douglas High School	February 14, 2018	Parkland, FL	17	17
Nashville Waffle House	April 22, 2018	Nashville, TN	4	4
Santa Fe High School	May 18, 2018	Santa Fe, TX	10	14
Cincinatti Fifth Third Center	September 6, 2018	Cincinnati, OH	4	2
Pittsburgh Synagogue	October 27, 2018	Pittsburgh, PA	11	7
Thousand Oaks Borderline Bar and Grill	November 7, 2018	Thousand Oaks, CA	13	16
Aurora Henry Pratt	February 15, 2019	Aurora, IL	6	7
Virginia Beach Municipal	May 31, 2019	Virginia Beach, VA	13	4
Gilroy Garlic Festival	July 28, 2019	Gilroy, CA	4	19
El Paso Walmart	August 3, 2019	El Paso, TX	23	23
Dayton Ned Peppers Bar	August 4, 2019	Dayton, OH	10	27
Springfield MO Kum and Go	March 15, 2020	Springfield, MO	5	2
Don Carter Lanes	December 26, 2020	Rockford, IL	3	3
Atlanta Spas	March 16, 2021	Atlanta, GA	8	1
Boulder King Soopers	March 22, 2021	Boulder, CO	10	2
Rock Hill Bar	April 7, 2021	Rock Hill, SC	6	0
Indianapolis Fed Ex	April 15, 2021	Indianapolis, IN	9	7
San Jose VTA	May 26, 2021	San Jose, CA	10	0
Collierville Kroger	September 23, 2021	Collierville, TN	2	14
Oxford High School	November 30, 2021	Oxford, MI	4	7
Garland TX Texaco	December 26, 2021	Garland, TX	3	1

Note: gray bars indicate mass shootings that we treat as chain shootings.

1.3 Sample Tweets

Figure A2: Sample of Tweets Following Stoneman Douglas Shooting

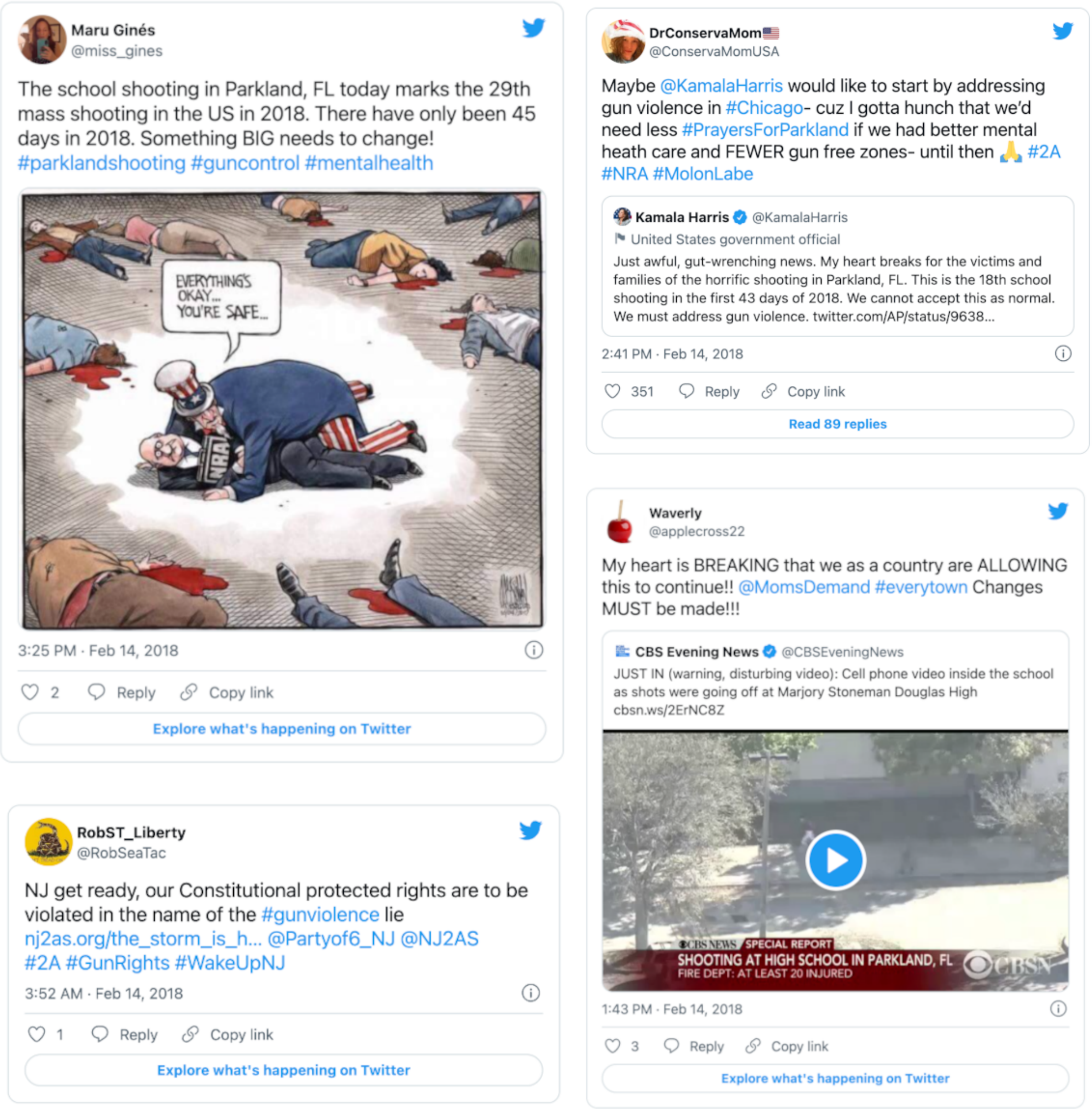

Note: Sample gun control and gun rights tweets following the Stoneman Douglas Shooting on February 14, 2018.

1.4 Lower Media Attention Shootings

Figure A3: Lower Media Attention Shootings and Google Search Behavior

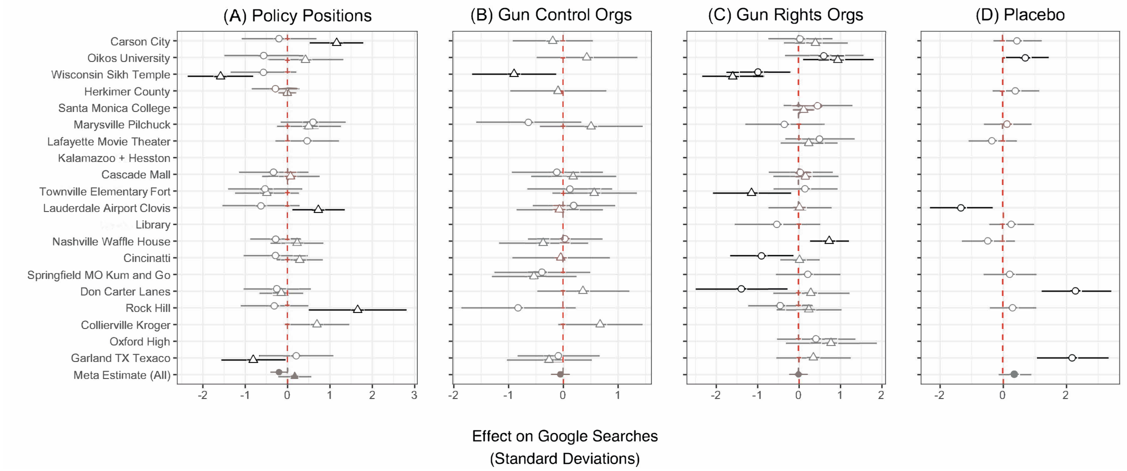

Note: RDiT treatment effect estimates with 95% confidence intervals. In Panel A circles indicate "Gun Control" and triangles "Gun Rights" searches. In Panel B, circles indicate "Everytown for Gun Safety" and triangles "Brady Campaign" searches. In Panel C circles indicate "Gun Owners of America" and triangles "NRA" searches. In panel D, estimates are for "recycling" searches. Grey point estimates indicate that the CIs includes 0. Missing estimates arise when there is no overlap between the time series outcome variable we measure and when a shooting occurred or insufficient data to estimate an effect.

Figure A4: Lower Media Attention Shootings and Documentary Streams

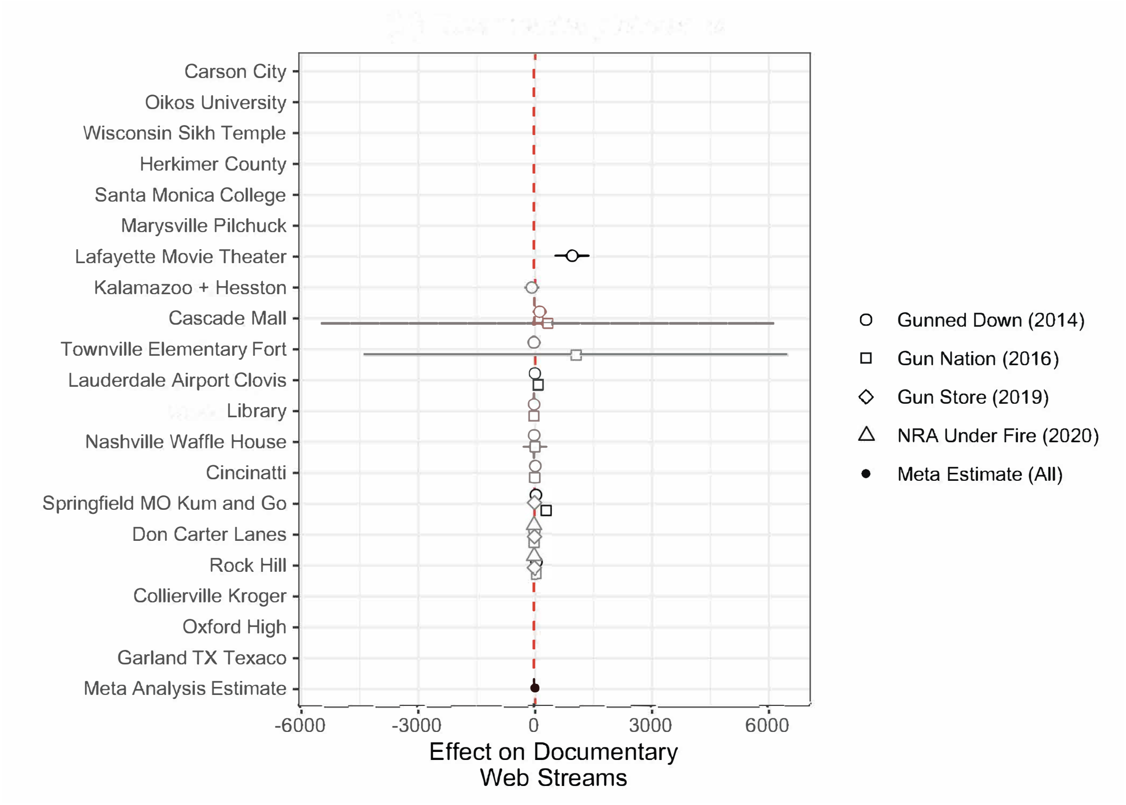

Note: RDiT treatment effect estimates with 95% confidence intervals. Grey point estimates indicate that the CIs includes 0. Missing estimates arise when there is no overlap between the time series outcome variable we measure and when a shooting occurred or insufficient data to estimate an effect.

Figure A5: Lower Media Attention Shootings and Tweets

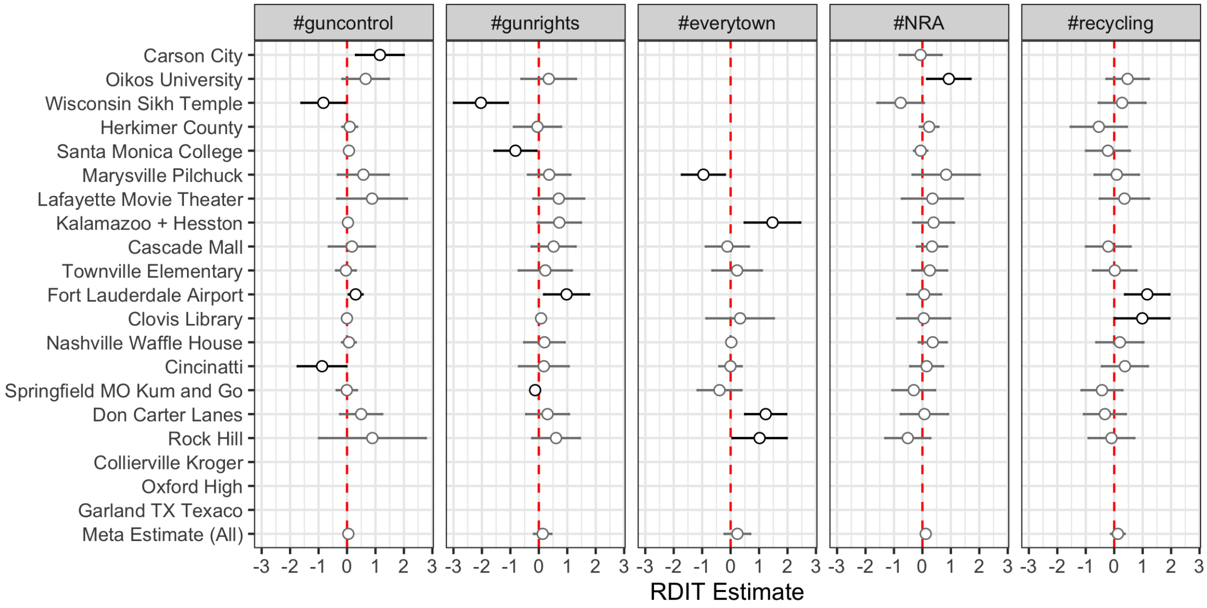

Note: RDiT treatment effect estimates with 95% confidence intervals. Grey point estimates indicate that the CIs includes 0. Missing estimates arise when there is no overlap between the time series outcome variable we measure and when a shooting occurred or insufficient data to estimate an effect.

Figure A6: Lower Media Attention Shootings and Petition Signatures

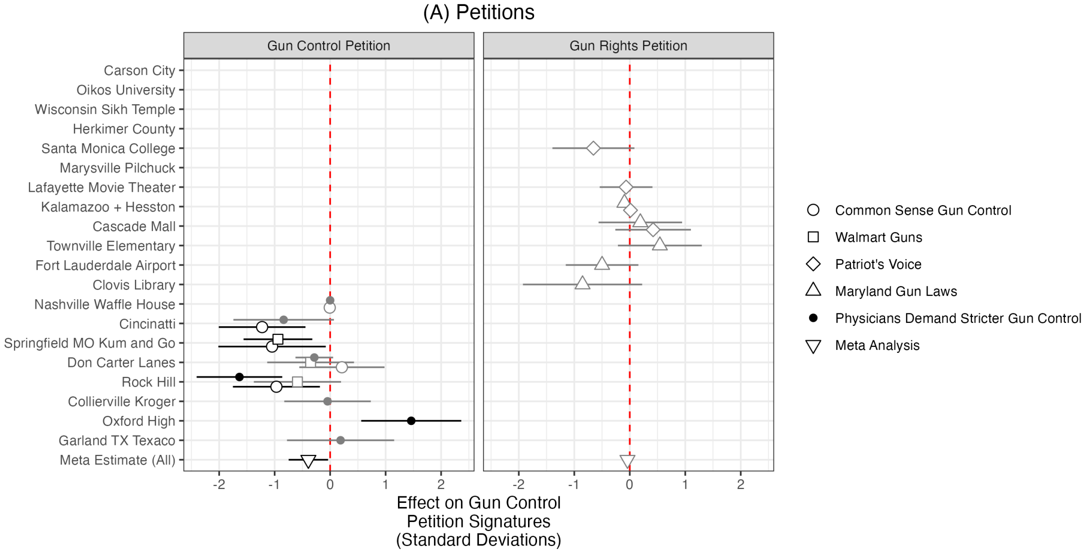

Note: RDiT treatment effect estimates with 95% confidence intervals. Grey point estimates indicate that the CIs includes 0. Missing estimates arise when there is no overlap between the time series outcome variable we measure and when a shooting occurred or insufficient data to estimate an effect.

Figure A7: Lower Media Attention Shootings and Donations

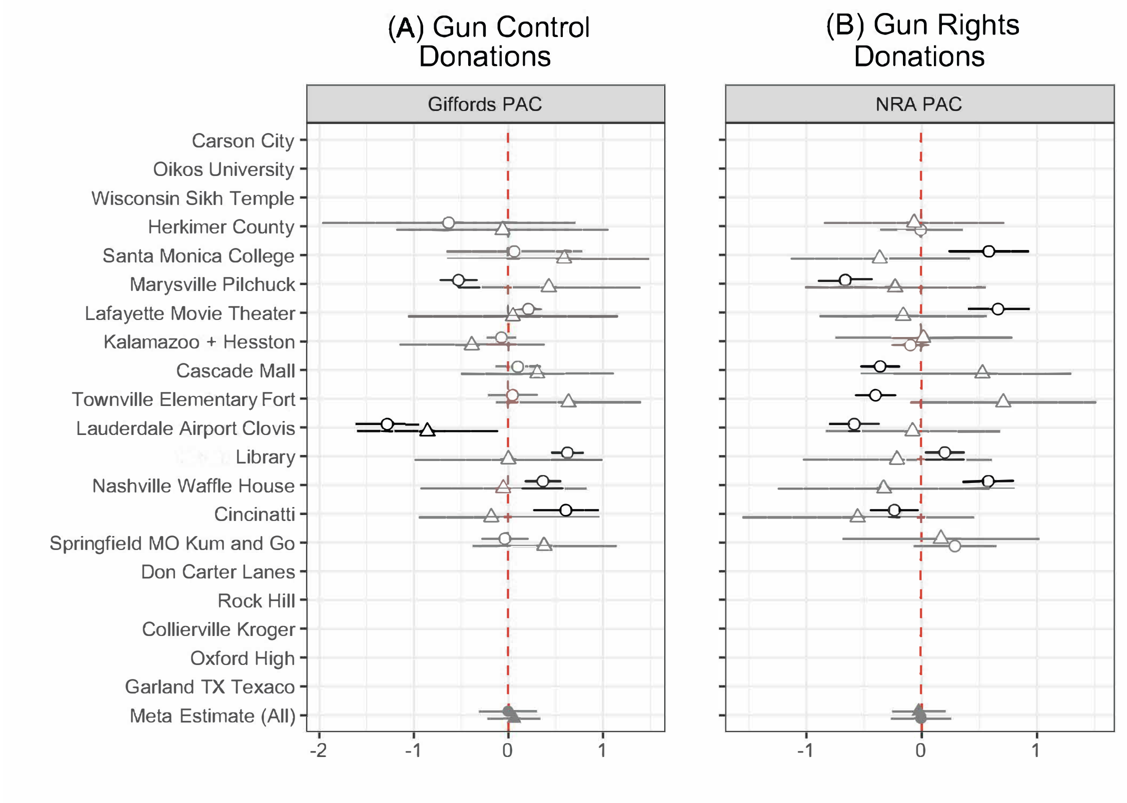

Note: RDiT treatment effect estimates with 95% confidence intervals. Grey point estimates indicate that the CIs includes 0. Missing estimates arise when there is no overlap between the time series outcome variable we measure and when a shooting occurred or insufficient data to estimate an effect.

2 Appendix B

2.1 Heterogeneous Treatment Effects By Proximity

Does proximity to a shooting moderate its effects on behavior? We may assume that those who live closer to a mass shooting will partake in a greater number of behaviors in the wake of the event than those who live further away (Newman and Hartman, 2017). To estimate the moderating effects of proximity, we re-ran our models for all outcomes for which we had geo-identifiers, estimating effects first for those living within a state and again for those living outside of a state. The outcomes for which we had geographic location include donation numbers and amounts to the NRA and Giffords PACs and petition signings for two Change.org gun control petitions: 1) “Physicians Demand Stricter Gun Control”; and 2) “Stop the sale of guns at Walmart stores.”

For both sets of outcomes we conducted two sets of analyses in order to compare the RDiT effect of mass shootings on in-state versus out-of-state behaviors. We wanted to investigate whether proximity to mass shootings (in-state) resulted in larger spikes in post-shooting behaviors compared to those further away (out-of-state). After running our RDiT models on split samples of the data we estimate the pooled meta-analytic average effect across outcomes for in-state versus out-of-state individuals. In Figures A8 and A9, we find no discernible differences in the magnitude of the treatment effects for those living closer to a shooting compared to those living further away for any of our donation or petition outcomes.

Figure A8: Effect of Shootings on In-State Versus Out-of-State Petitions

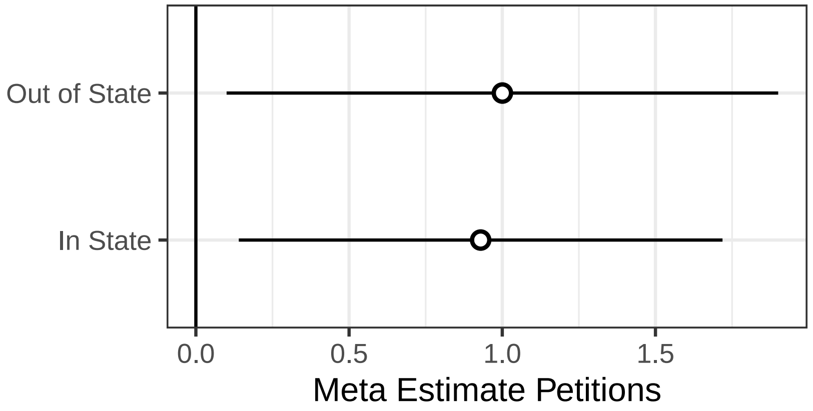

Note: Pooled RDiT treatment effect estimates with 95% confidence intervals.

Figure A9: Effect of Shootings on In-State Versus Out-of-State Donations

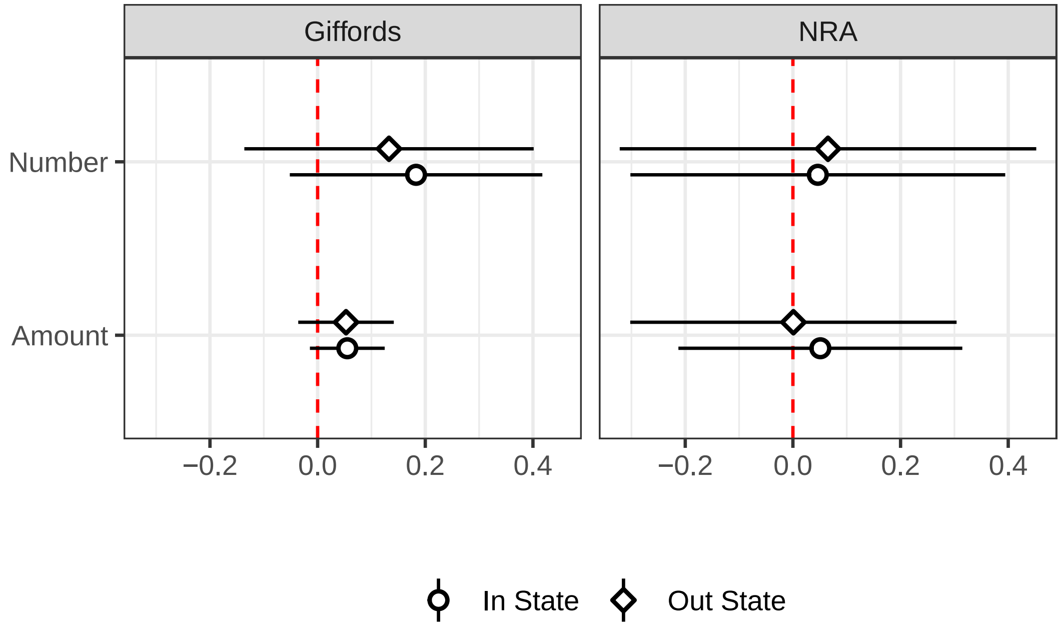

Note: Pooled RDiT treatment effect estimates with 95% confidence intervals.

2.2 Heterogeneous Treatment Effects By Race of Victim

It may also be the case that Americans react with greater numbers of behaviors to shootings where a greater proportion of the victims are white. Research finds, for example, that shootings where most of the victims are black generate less sympathy and policy discourse than those shootings where most of the victims are white (Zhang et al., 2019).

To estimate the moderating effects of race-of-victims on our outcomes we first had to code the race of victims for all the mass shootings for which we had data. To do so, we first used Google searches to build a database of each victim's name. We then used the R package rethnicity to estimate the race and ethnicity of victims based on their first name and surname (Xie, 2022). The package provides a probabilistic estimate of race and ethnicity across four categories: White, Black, Latino, and Asian. For some respondents, the estimate was extremely precise. Xiaojie Tan, for example, a victim of the 2021 Atlanta Spa mass shooting was estimated to be Asian American with 99.9% certainty. Others were far less certain. Alexander Boik of the 2012 Aurora Movie Theater shooting had a 22.6% probability of being Asian, a 9.4% probability of being Black, a 15% probability of being Latino, and a 53% probability of being White. In these cases where there was no clear consensus estimate (for our purposes we deemed 75% probability and higher as being a clear consensus estimate), we conducted an additional Google search and tried to validate the prediction using images and/or news stories on the victims. For clear cases where the estimate was wrong (e.g. Black versus White), we updated the coding based on our additional research. For cases where the search provided little additional information, we relied on the probabilistic estimate from the package. Across all 436 victims in the shootings we study, we estimated that 59% were non-Hispanic white, 9% were Black, 22% were Hispanic,

and 10% were Asian. When we aggregate up to the shooting level, we find that the percent of victims who were white range from 100% (9 shootings) to 0% (6 shootings). The average percent white across the shootings was 62%.

Figure A10: Race of Victim and Media Coverage

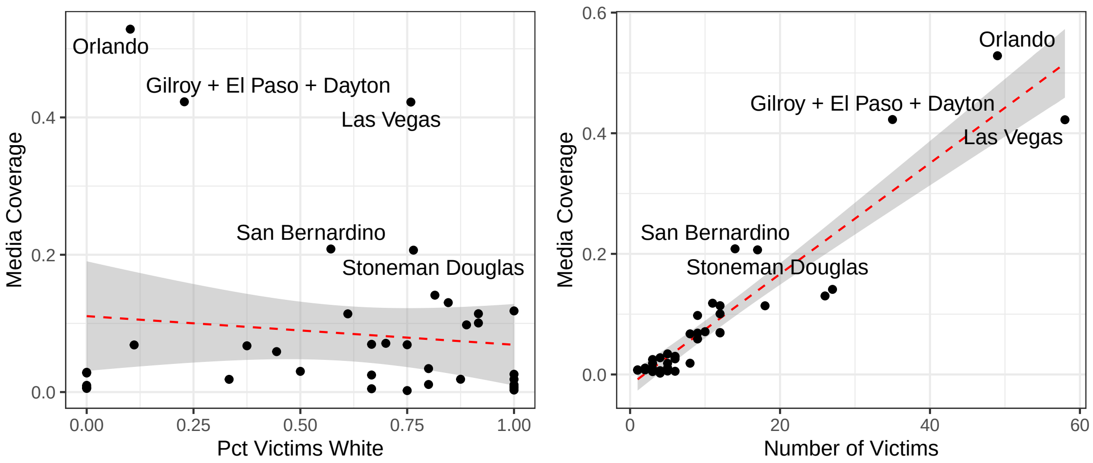

Note: the relationship between the percentage of victims who are white in each shooting and media coverage (panel A) and number of victims and media coverage (panel B) across all shootings in our dataset.

Before we estimated heterogeneous treatment effects, we first examined the relationship between the proportion of victims in the shooting who were white and media coverage of the shooting using our measure of media coverage. In the first panel of Figure A10 we show first that there is no relationship between the percentage of the victims who are white and media coverage of that shooting. Instead, as is consistent with the literature (Schildkraut et al., 2018), we find that media coverage is very strongly related to number of victims, as we show in the second panel.

While this casts doubt on the hypothesis that effects may vary as a function of the race of victims, we nevertheless replicate Panel A of Figure 8—a meta-analysis of effects from our Google Trends, Twitter, petitions, and donations—but pooling effects from shootings by tercile of percentage of the victims who are white. As we show in Figure A11, there appears to be no consistent discernible difference in treatment effects across shootings as a function of percentage of the victims who are white. In fact, the only statistically significant differences appear to be smaller effects for shootings with more white victims relative to those with more non-white victims in our donations data, though these estimates are imprecise and are themselves indistinguishable from zero.

Figure A11: Race of Victims and Treatment Effect Heterogeneity

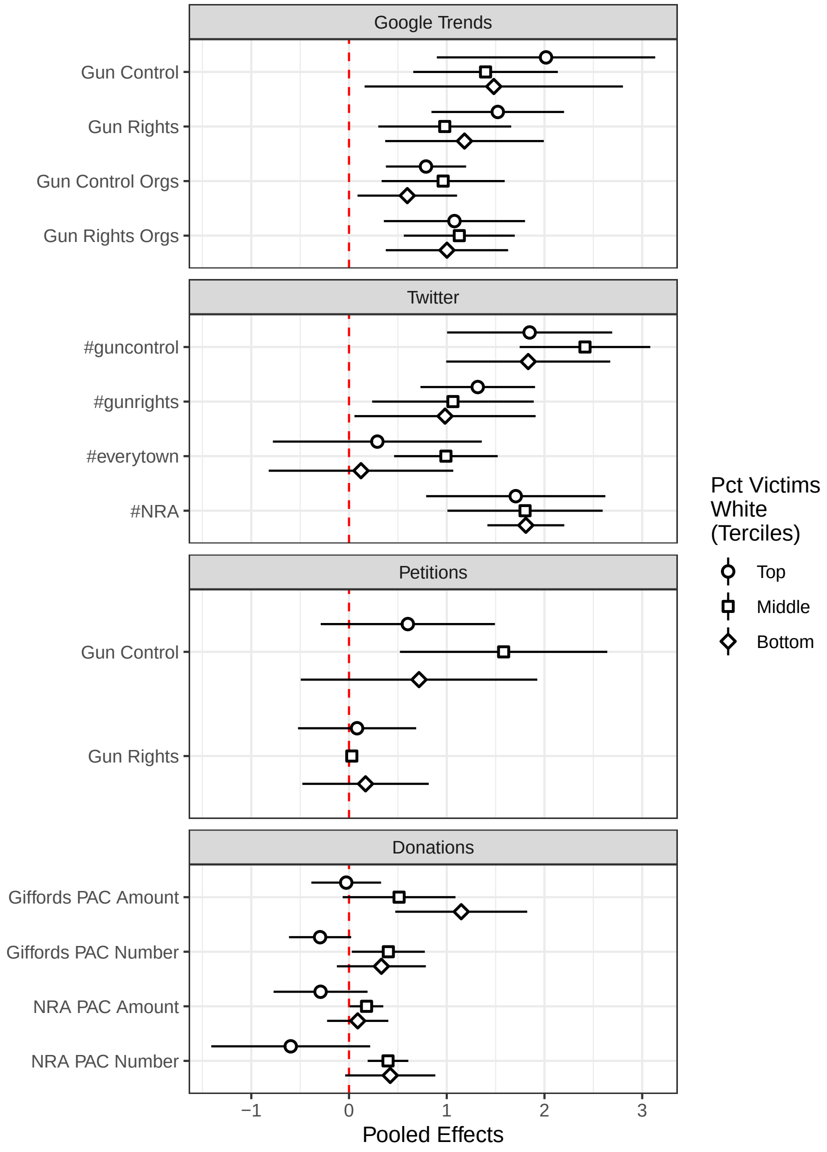

Note: pooled RDiT treatment effect estimates with 95% confidence intervals.

References

Hausman, C. and D. S. Rapson (2018). Regression Discontinuity in Time: Considerations for Empirical Applications. *Annual Review of Resource Economics 10*, 533–552.

Imbens, G. and K. Kalyanaraman (2012). Optimal bandwidth choice for the regression discontinuity estimator. *79*, 933–959.

Newman, B. J. and T. K. Hartman (2017). Mass shootings and public support for gun control. *49*, 1527–1553.

Schildkraut, J., H. J. Elsass, and K. Meredith (2018). Mass shootings and the media: why all events are not equal. *41*, 223–243.

Stigler, M. and B. Quast (2016). rddtools: A toolbox for regression discontinuity in r. Technical report, The Graduate Institute, Maison de la paix, Geneva, Switzerland.

Wing, C. and T. D. Cook (2013). Strengthening the Regression Discontinuity Design Using Additional Design Elements: A Within-Study Comparison. *Methods of Policy Analysis 32*(4), 853–877.

Xie, F. (2022). rethnicity: An r package for predicting ethnicity from names. *17*, 1–5.

Zhang, Y., D. Shah, J. Foley, A. Abhishek, J. Lukito, J. Suk, S. J. Kim, Z. Sun, J. Pevehouse, and C. Garlough (2019). Whose lives matter? mass shootings and social media discourses of sympathy and policy, 2012–2014. *24*, 182–202.
